# Observation of giant and tunable thermal diffusivity of a Dirac fluid at room temperature

**DOI:** 10.1038/s41565-021-00957-6

**Published:** 2021-08-23

**Authors:** Alexander Block, Alessandro Principi, Niels C. H. Hesp, Aron W. Cummings, Matz Liebel, Kenji Watanabe, Takashi Taniguchi, Stephan Roche, Frank H. L. Koppens, Niek F. van Hulst, Klaas-Jan Tielrooij

**Affiliations:** 1 ICFO (Institut de Ciències Fotòniques), The Barcelona Institute of Science and Technology, Castelldefels, Spain; 2 Catalan Institute of Nanoscience and Nanotechnology (ICN2), BIST and CSIC, Bellaterra, Spain; 3 School of Physics and Astronomy, University of Manchester, Manchester, UK; 4 Research Center for Functional Materials, National Institute for Materials Science, Tsukuba, Japan; 5 International Center for Materials Nanoarchitectonics, National Institute for Materials Science, Tsukuba, Japan; 6 ICREA - Institució Catalana de Recerca i Estudis Avançats, Barcelona, Spain

**Keywords:** Graphene, Electronic properties and materials, Ultrafast photonics

## Abstract

Conducting materials typically exhibit either diffusive or ballistic charge transport. When electron–electron interactions dominate, a hydrodynamic regime with viscous charge flow emerges^1–13^. More stringent conditions eventually yield a quantum-critical Dirac-fluid regime, where electronic heat can flow more efficiently than charge^14–22^. However, observing and controlling the flow of electronic heat in the hydrodynamic regime at room temperature has so far remained elusive. Here we observe heat transport in graphene in the diffusive and hydrodynamic regimes, and report a controllable transition to the Dirac-fluid regime at room temperature, using carrier temperature and carrier density as control knobs. We introduce the technique of spatiotemporal thermoelectric microscopy with femtosecond temporal and nanometre spatial resolution, which allows for tracking electronic heat spreading. In the diffusive regime, we find a thermal diffusivity of roughly 2,000 cm^2^ s^−1^, consistent with charge transport. Moreover, within the hydrodynamic time window before momentum relaxation, we observe heat spreading corresponding to a giant diffusivity up to 70,000 cm^2^ s^−1^, indicative of a Dirac fluid. Our results offer the possibility of further exploration of these interesting physical phenomena and their potential applications in nanoscale thermal management.

## Abstract (web summary)

Spatiotemporal thermoelectric microscopy enables the observation of electronic heat flow in graphene in diffusive and hydrodynamic regimes at room temperature, as well as a controlled transition from a Fermi liquid to Dirac fluid.

## Main

During the last few years, signatures of viscous charge flow in the so-called Fermi-liquid hydrodynamic regime were observed in two-dimensional (2D) electron systems, especially graphene, using electrical device measurements^7–9,11,12^ and scanning probe microscopy^10,13,22^. A second hydrodynamic regime, which has no analogue in classical fluids, can occur very close to the Dirac point. When the Fermi temperature (*T*_F_ *=* *E*_F_/*k*_B_, where *E*_F_ is the Fermi energy and *k*_B_ is the Boltzmann constant) becomes small compared to the electron temperature *T*_e_, the system becomes a quantum-critical fluid^3,6,14,15,17^. In this Dirac-fluid regime, the non-relativistic description of the viscous fluid is replaced by its ultra-relativistic counterpart, which accounts for the presence of both particles and holes, as well as for their linear energy dispersion. In line with theoretical predictions in this regime^15^, electrical measurements at cryogenic temperatures indicated a deviation from the Wiedemann–Franz law^19^ and from the Mott relation^20^, and a terahertz-probe study revealed the quantum-critical carrier scattering rate^21^.

**Fig. 1:**
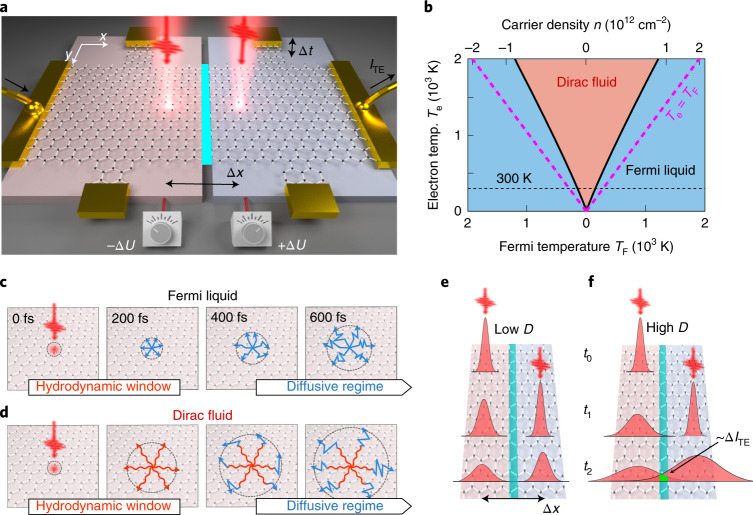
Spatiotemporal thermoelectric microscopy and heat spreading regimes. **a**, Concept of the experiment, where a graphene Hall bar and thermoelectric device is illuminated by two femtosecond heat-generating pulses with a relative temporal offset ∆*t* and a symmetric spatial offset ∆*x* with respect to the pn junction where electronic heat generates a thermoelectric current. The junction is created by applying +∆*U* to one gate and −∆*U* to the other. We isolate the differential thermoelectric current corresponding to light-induced electronic heat from both pulses that has travelled to the junction, where the heat adds up in a non-linear fashion. **b**, Phase diagram of the Dirac-fluid regime, calculated following ref. ^14^. For increasing *T*_e_, the Dirac-fluid regime occurs increasingly far away from the Dirac point. **c**,**d**, Illustration of light-triggered spreading of electronic heat in the Fermi-liquid regime (**c**) and Dirac-fluid regime (**d**). In both cases, for Δ*t* > *τ*_mr_, diffusive transport dominates (straight blue lines), while in the hydrodynamic window, with Δ*t* < *τ*_mr_, extremely efficient heat transport occurs in the Dirac-fluid regime (wavy red lines). **e**,**f**, Sketch of the spatial broadening of the heat spots for low (**e**) and high (**f**) diffusivity, *D*, indicating more interacting heat at the junction region, hence higher ∆*I*_TE_ signal for higher *D*.

**Extended Data Fig. 1:**
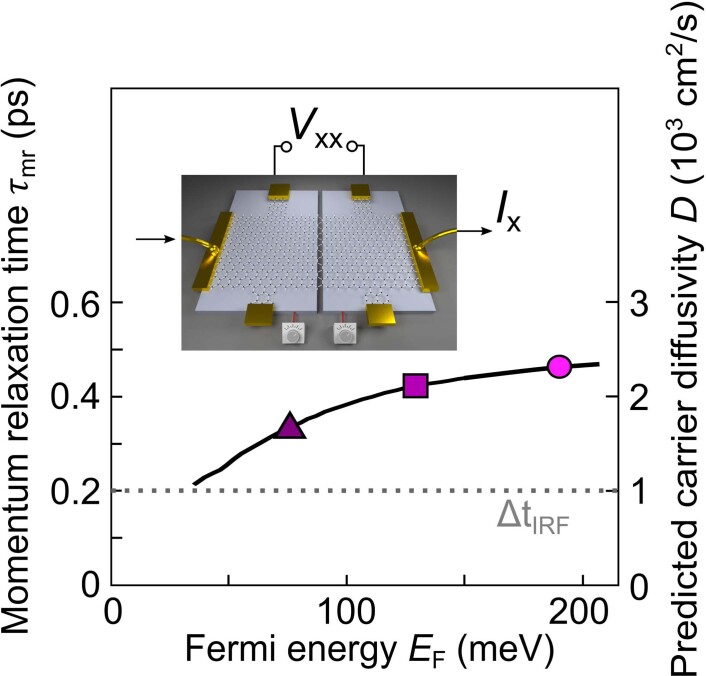
Electrical mobility measurement. Momentum relaxation time from four-probe measurements and corresponding (calculated) heat diffusivity (solid line). Four-probe measurements were performed by applying 1 V to a MΩ series resistor, such that a current of 1 μA flows between the two outer contacts of the device (see inset). We then measure the voltage drop between two lateral contacts of the graphene device. This yields the sheet conductance σ as a function of gate voltage. We then use σ = *ne*μ, in order to extract the mobility µ and use $\tau _{mr} = \frac{{\mu E_{{{\mathrm{F}}}}}}{{ev_{{{\mathrm{F}}}}^2}}$ to obtain the momentum relaxation time. Here, *n* is the carrier density, *E*_F_ is the Fermi energy, *v*_F_ ≈ 10^6^ m/s is the Fermi velocity, and *e* is the elementary charge. We measured up to *E*_F_ = 150 meV, and extrapolated the data to higher Fermi energies. The three symbols indicate the Fermi energy and predicted diffusivity corresponding to the diffusivity measurements in Fig. 2e.

Here, we follow electronic heat flow in the diffusive and hydrodynamic regimes at room temperature, and demonstrate a controlled Fermi-liquid to Dirac-fluid crossover, with a strongly enhanced thermal diffusivity close to the Dirac point. These observations are enabled by ultrafast spatiotemporal thermoelectric microscopy, a technique inspired by all-optical spatiotemporal diffusivity measurements^23–25^, with the crucial difference that the observable is the thermoelectric current, which is directly, and exclusively, sensitive to electronic heat^26^. We use a hexagonal boron nitride (hBN)-encapsulated graphene device that is both a Hall bar for electrical measurements and a split-gate thermoelectric detector (Fig. 1a). Since we use ultrashort laser pulses, with an approximate instrument response time (Δ*t*_IRF_ where IRF means instrument response function) of 200 fs, to generate electronic heat, we are able to examine the system before momentum relaxation occurs, as we measure a momentum relaxation time, *τ*_mr_, around 350 fs (Extended Data Fig. 1). In this temporal regime before momentum is relaxed, we enter the hydrodynamic window, because the electron–electron scattering time *τ*_ee_ is <100 fs (ref. ^27^), that is *τ*_ee_ < Δ*τ*_IRF_ < *τ*_mr_. This is a different approach compared to most previous studies, where hydrodynamic effects were observed by using small system dimensions *L* to eliminate effects of momentum relaxation, that is *v*_F_*τ*_ee_ < *L* < *v*_F_*τ*_mr_ (refs. ^7–13,19,22^) (*v*_F_ = 10^6^ m s^−1^ is the Fermi velocity). Our approach furthermore exploits elevated carrier temperatures, which greatly increases the accessibility of the Dirac-fluid regime, as for increasing carrier temperatures the crossover occurs increasingly far away from the Dirac point^14,17^ (Fig. 1b). As we will show, during the hydrodynamic window substantially more efficient heat spreading occurs in the Dirac-fluid regime than in the Fermi-liquid regime and in the diffusive regime (Fig. 1c,d).

Our technique works by using two ultrafast laser pulses that produce localized spots of electronic heat within tens of femtoseconds^27^. These spots are characterized by an increased carrier temperature *T*_e_ > *T*_l_, with *T*_l_ the lattice temperature (300 K). The degree of spatial spreading of these electronic heat spots as a function of time is governed by the diffusivity *D*. We control the relative spatial and temporal displacement, Δ*x* and Δ*t*, of the two pulses with sub-100-nm spatial precision and roughly 200 fs temporal resolution. Each laser pulse is incident on opposite sides of a pn junction at a distance ∆*x*/2 from the junction. This pn junction is created by applying opposite voltages ±Δ*U* with respect to the Dirac point voltages to the two backgates that form a split-gate structure. The two photo-generated electronic heat spots spread out spatially and part of the heat can reach the pn junction after a certain amount of time, generating a thermoelectric current at the junction through the Seebeck gradient^26^. The small region of the pn junction thus serves as a local probe of the electron temperature. While each of the heat spots can create thermoelectric current independently, we obtain spatiotemporal information by examining exclusively the signal that corresponds to heat generated by one of the pulses interacting with heat generated by the other pulse—the interacting heat current, Δ*I*_TE_. Since the thermoelectric photocurrent scales sublinearly with incident power, we can isolate this interacting heat current Δ*I*_TE_ by modulating each laser beam at a different frequency, *f*_1_ and *f*_2_, and demodulating the thermoelectric current at the difference frequency *f*_1_ − *f*_2_. As illustrated in Fig. 1e,f, the higher the diffusivity *D*, the more interacting heat current Δ*I*_TE_ remains for increasing Δ*x* and Δ*t*.

**Fig. 2:**
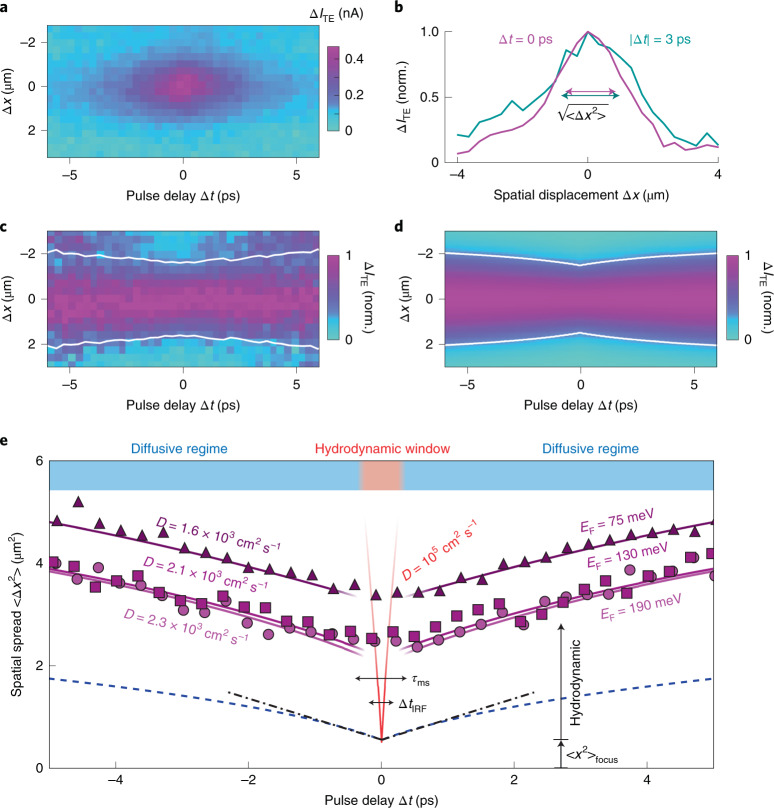
Spatiotemporal tracking of heat spreading. **a**, The experimental spatiotemporal differential thermoelectric current ∆*I*_TE_ as a function of Δ*x* and Δ*t*. **b**, Normalized profiles (norm.), showing a larger spatial spread for larger |∆*t*|. **c**,**d**, Experimental (**c**) and simulated (**d**) normalized ∆*I*_TE_ for each ∆*t*, showing spatial broadening due to thermal transport as a function of ∆*t*. The white line indicates the spatial spread <∆*x*^2^>. **e**, Spatial spread <∆*x*^2^> of ∆*I*_TE_, as a function of ∆*t* for three different Fermi energies (symbols), with simulation results using as input the diffusivities from electrical mobility measurements (purple solid lines), with offset due to ultrafast heat spreading around time zero. Simulation (blue dashed line) and theoretical heat equation (black dash-dotted line) results with the same input diffusivity and no ultrafast spreading around time zero. Heat spreading with ultrahigh diffusivity in the Dirac-fluid regime (red line), which lasts for a few hundred fs, explains the time zero offset.

Figure 2a shows the measured interacting heat current ∆*I*_TE_ as a function of Δ*x* and Δ*t*. As expected, the largest ∆*I*_TE_ occurs for the largest spatiotemporal overlap at the pn junction (Δ*x* = Δ*t* = 0). For increasing |∆*t*|, we find that the normalized signal extends further spatially, indicating the occurrence of heat spreading (Fig. 2b). This spatial spread is quantified via the second moment <Δ*x*^2^>, which quantifies the width of the profile at different time delays (Methods). Similar to recent all-optical spatiotemporal microscopy^24,25^, we obtain spatial information beyond the diffraction limit by precise spatial sampling of diffraction-limited profiles. The experimentally obtained spatial spread as a function of Δ*t* (Fig. 2c) is very similar to the calculated results (Fig. 2d), obtained by simulating the experiment with a given diffusivity, *D* (Methods and Supplementary Note 1). The white lines indicate the values of the spatial spread <Δ*x*^2^> for different Δ*t*. We also compare the simulated spatial spread <Δ*x*^2^> versus Δ*t* (blue dashed line in Fig. 2e) with the theoretical expectation according to the heat diffusion equation, <Δ*x*^2^> = <Δ*x*^2^>_focus_ + 2*D*Δ*t* (dash-dotted line in Fig. 2e). Here, *D* is the same diffusivity that was used as input for the simulation, and <Δ*x*^2^>_focus_ is the minimum second moment from the two overlapping pulses (Supplementary Note 2 and Supplementary Figs. 1–4). The initial slope is the same for both the simulated heat spreading and the theoretical spreading following the heat diffusion equation.

We first discuss the experimental results in the diffusive regime, where |Δ*t*| > *τ*_mr_. For three different gate voltage combinations, corresponding to Fermi energies between 75 and 190 meV (*T*_F_ = 900 − 2,200 K), we extract the spatial spread as a function of time delay (symbols in Fig. 2e), and compare it with the results from simulations (solid lines). For these simulations, we have used the diffusivity values that we obtain directly from electrical measurements of charge mobility *μ* on the same device (Extended Data Fig. 1), and the relation between mobility and diffusivity: *D* = *μE*_F_/2*e* (Methods). We find excellent agreement, if we account for short-lived ultrafast heat spreading around Δ*t* = 0, which leads to a larger-than-expected initial spread at time zero <Δ*x*^2^>_min_, as we will explain below. The agreement between the measured heat spread for |Δ*t*| > *τ*_mr_ and the one calculated using the measured charge mobilities shows that electronic heat and charge flow together, as expected in the diffusive regime. Furthermore, it confirms that our technique is a reliable method for obtaining thermal diffusivities in a quantitative manner.

**Extended Data Fig. 2:**
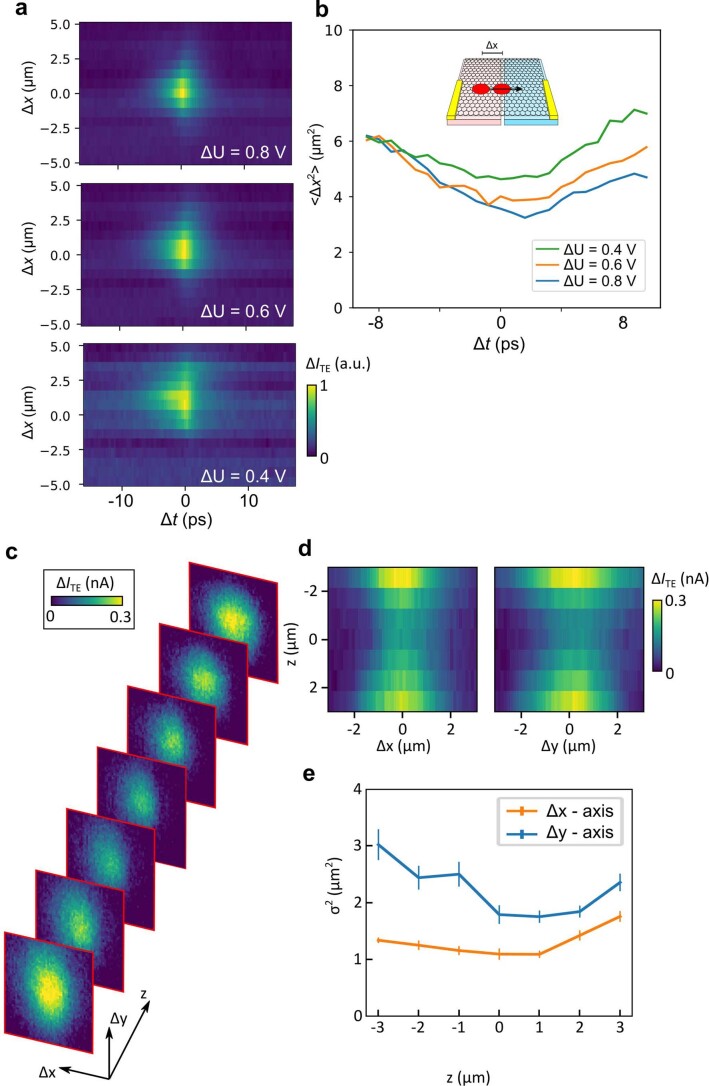
Spatiotemporal results from a second device. **a**, Asymmetric spatiotemporal ∆*I*_TE_ maps for three different gate voltages. **b**, Extracted width as a function of ∆*t*. A lower Fermi level leads to a higher time-zero width, in accordance with hydrodynamic transport, as presented for the main device in the manuscript. (**c**) ∆*I*_TE_ maps, taken at ∆*t* = 0, as a function of beam offset (∆*x*, ∆*y*), as well as sample height (*z*). (**d**) extracted line profiles for the two dimensions. **e**, Resulting signal width σ^2^ for both dimensions as extracted from Gaussian fits at each *z*-position. The same measurements are presented in Supplementary Fig. 4 for the main device. These experiments were performed with two beams of wavelength 443 nm and 886 nm, respectively.

**Extended Data Fig. 3:**
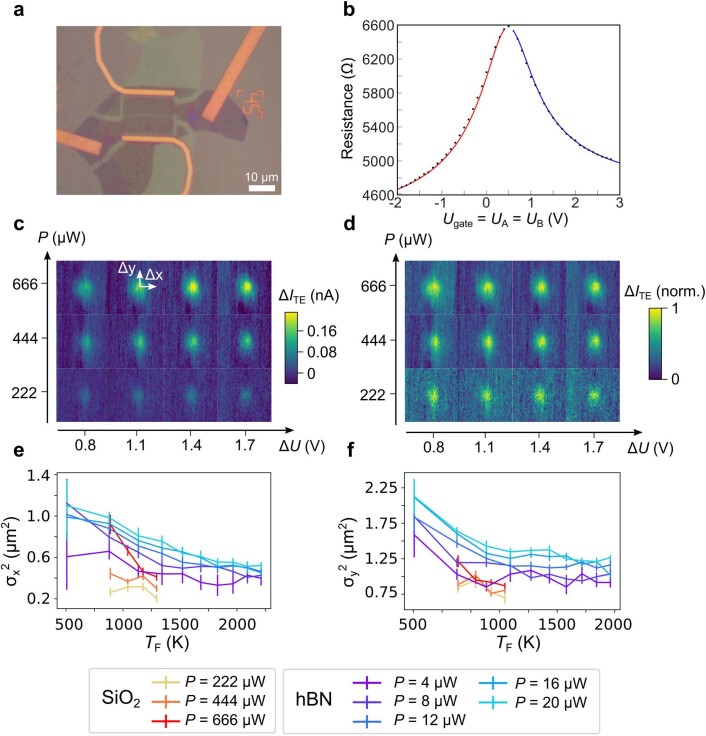
Spatiotemporal results from a third device. **a**, Microscope image of the device. This sample has split-gates made from graphite, with a gap that is 200 nm. It then has a 30 nm thick layer of SiO_2_, and then we transferred a graphene flake grown by chemical vapour deposition (CVD) on top of the split-gate structure using an hBN flake. **b**, Gate-dependent current measurement, which gives an estimated mobility of this half-encapsulated CVD graphene sample of around 8,500 cm^2^/Vs (solid lines). **c-d**, Asymmetric spatiotemporal ∆*I*_TE_ maps at time zero for different gate voltages and laser powers without (**c**) and with normalization (**d**). **e-f**, Comparison between first and third device. Time zero Gaussian widths for spatial scans with one pulse on the junction and the second one scanning across (**e**) and along (**f**) the graphene *pn*-junction, as a function of power and gate voltage, for both the first device (hBN-encapsulated with high mobility, presented in the main text, blue-purple colours, ‘hBN’) and the third device (on SiO_2_ with low mobility, yellow-red colors, ‘SiO_2_’). The low-mobility sample with shorter hydrodynamic time window shows systematically less heat spreading around time zero, in agreement with our picture of hydrodynamic heat spreading during the hydrodynamic time window.

We now turn to the non-diffusive regime, by exploring the behaviour in the hydrodynamic window, where |Δ*t*| < *τ*_mr_. The experimentally obtained spatial spreads start at a minimum value <Δ*x*^2^>_min_ larger than 2 μm^2^, rather than starting at an expected <Δ*x*^2^>_focus_ = 0.56 μm^2^. A second device of hBN-encapsulated graphene with similar mobility reproduces this larger-than-expected spatial spread at time zero (Supplementary Note 3 and Extended Data Fig. 2). We exclude the possibility of an experimental artefact such as an underestimation of the laser spot size, since we repeated the measurements while scanning through the laser focus, and measured the focus size (Supplementary Figs. 1–4). Furthermore, we observe that the offset depends on the Fermi energy, while keeping all other experimental parameters fixed. Finally, we measured a third device with a lower charge mobility and shorter hydrodynamic time window: *τ*_mr_ < 100 fs, which is smaller than Δ*t*_IRF_. This device exhibits systematically less heat spreading around time zero (Supplementary Note 4 and Extended Data Fig. 3), consistent with its smaller hydrodynamic time window. We therefore attribute the large experimentally observed minimum <Δ*x*^2^>_min_ in Fig. 2e to ultrafast initial heat spreading that occurs before momentum relaxation takes place, Δ*t* ≲ 350 fs (see the schematic illustration of spatiotemporal heat spreading in Fig. 1d). The dynamics of this initial heat spreading are washed out by the finite time resolution Δ*t*_IRF_, and manifests as a large minimum <Δ*x*^2^>_min_ at time zero. The observed initial spatial spread suggests a thermal diffusivity of *D* = (<Δ*x*^2^>_min_ − <Δ*x*^2^>_focus_)/2Δ*t*_IRF_ ≅ 70,000 cm^2^ s^−1^ for the lowest measured *E*_F_ of 75 meV. Simulations of heat spreading with an input diffusivity of 100,000 cm^2^ s^−1^ are indeed consistent with the experimentally observed spread in the hydrodynamic window (the red line in Fig. 2e).

**Extended Data Fig. 4:**
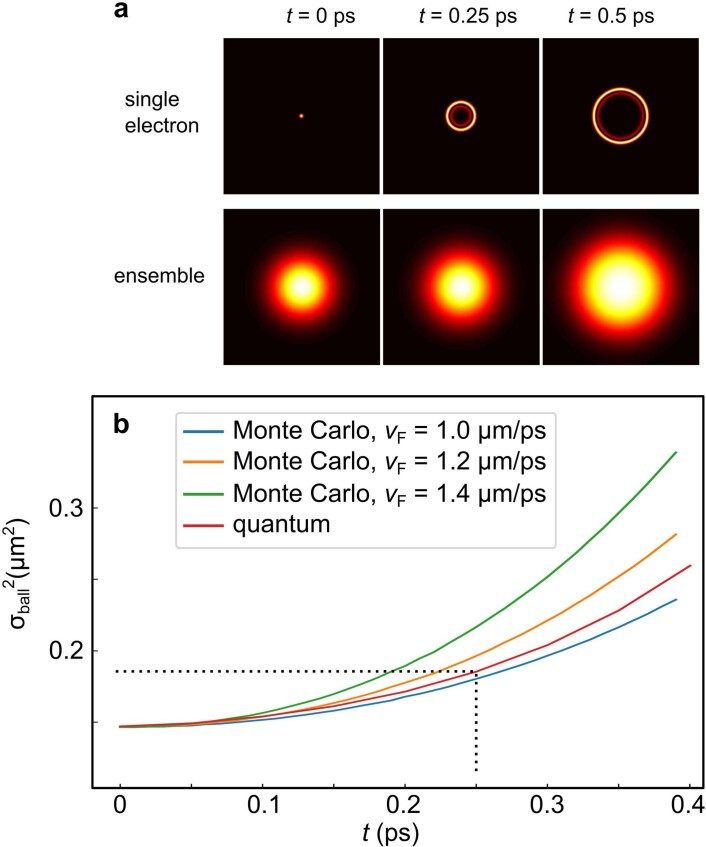
Ballistic spreading simulation. **a**, Time-dependent output distributions for quantum mechanical calculations for a single electron (top row) and an ensemble of independent electrons (bottom row). **b**, Resulting width σ_ball_^2^ for ballistic transport for the Monte Carlo method with varying Fermi velocities, as well as the quantum calculation for an ensemble of independent electrons. Both calculations essentially agree and the spread within 0.25 ps leads to final widths of below 0.25 µm^2^ for realistic values of the Fermi velocity.

We attribute this observation of highly efficient initial heat spreading to the presence of the quantum-critical electron-hole plasma. We can exclude that the observed initial spreading is the result of ballistic transport, as we calculate that the ballistic contribution to initial heat spreading would give only <Δ*x*^2^>_ball_ = 0.68 μm^2^ (Supplementary Note 2 and Extended Data Fig. 4). Besides, ballistic transport has a very weak dependence (<10%) on carrier density in this range, as the Fermi velocity does not change appreciably for the Fermi energies considered here^28^. The reason for the high diffusivity in the Dirac-fluid regime is that the hot electrons and hot holes that coexist in this regime move in the same direction under a thermal gradient, with inter-particle scattering events conserving total momentum^19^. We note that typical transport measurements probe the sum of the momentum-conserving thermal resistivity (due to carrier–carrier scattering) and the momentum-non-conserving thermal resistivity (due to carrier–impurity and carrier–phonon scattering), where the latter dominates at room temperature. The ability of our technique to interrogate the system during the 350 fs before any momentum-non-conserving scattering occurs, means that this contribution to the overall resistivity is negligible. Therefore, we probe exclusively the momentum-conserving thermal conductivity, which diverges towards the Dirac point and towards infinite electron temperature.

**Fig. 3:**
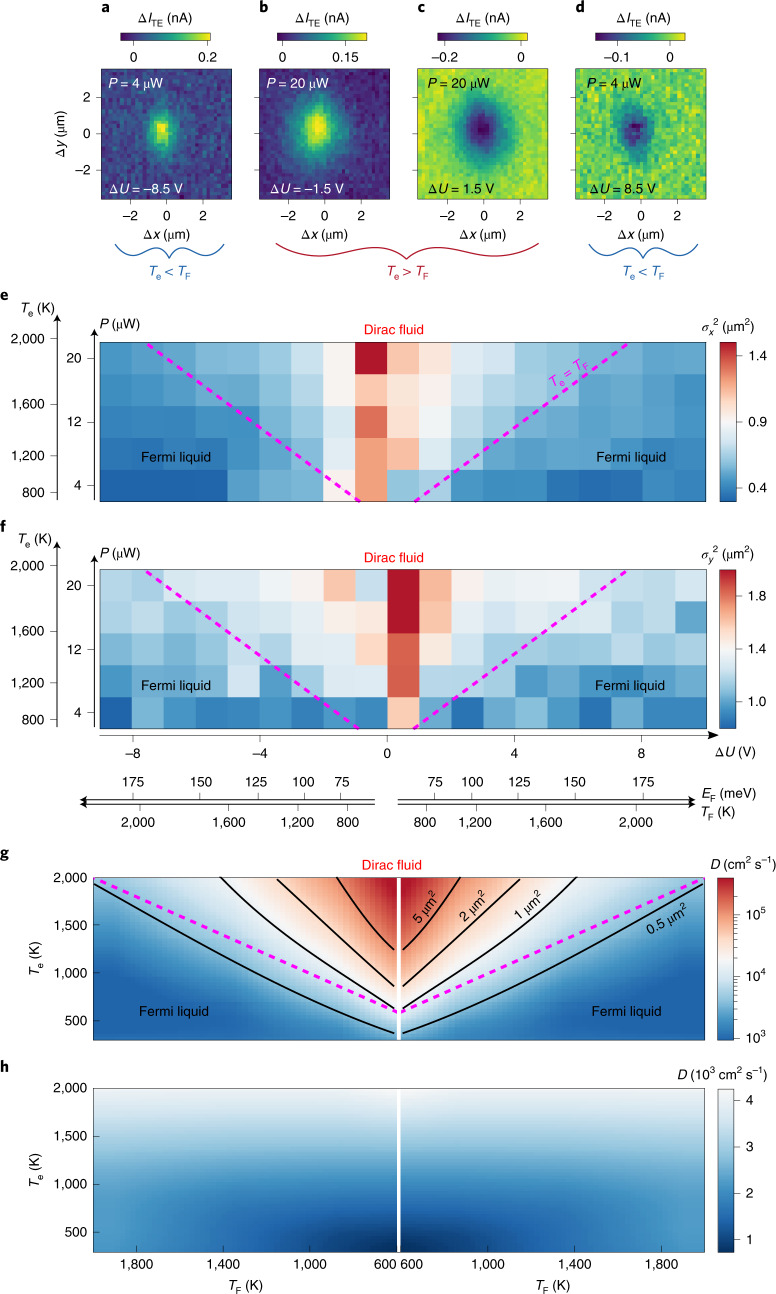
Fermi-liquid to Dirac-fluid crossover. **a**–**d**, Time-zero spatial maps of ∆*I*_TE_ for low optical power *P* and high gate voltage ∆*U* with an np junction (**a**) and with a pn junction (**d**); and for high optical power and low gate voltage with an np junction (**b**), and with a pn junction (**c**). For larger ratio *T*_e_/*T*_F_ (that is, larger *P*/∆*U*) the spatial extent is clearly larger. **e**,**f**, Time zero Gaussian widths for spatial scans with one pulse on the junction and the second one scanning across (**e**) and along (**f**) the graphene pn junction, as a function of *P* and ∆*U*. The strong dependence on *T*_e_/*T*_F_ demonstrates our ability to transition under control into the Dirac-fluid regime with strongly increased thermal diffusivity. **g**,**h**, Calculation of the thermal diffusivity following refs. ^6,18^ with only electron–electron interactions (**g**) and only long-range Coulomb scattering (**h**). The contours in **g** are the calculated time zero spreads $\sigma _{{{{\mathrm{calc}}}}}^2$ (Methods).

To provide further evidence of hydrodynamic heat transport, we demonstrate the ability to control the crossover between the Fermi-liquid and quantum-critical Dirac-fluid regimes via the ratio *T*_e_/*T*_F_, by independently varying *T*_e_ via the incident laser power and *T*_F_ via the applied gate voltage. A larger ratio results in less Coulomb screening and correspondingly stronger hydrodynamic effects due to electron–electron interactions. If *T*_e_ is larger than *T*_F_, electrons and holes coexist and the Dirac-fluid regime becomes accessible (Fig. 1b). We perform spatial scans in the hydrodynamic window at a temporal delay of Δ*t* = 0, in a geometry with one laser pulse impinging on the junction, while scanning the other pulse across (*x* axis) and along (*y* axis) the junction region. Figure 3a–d shows four representative spatial ∆*I*_TE_ maps with varying *T*_e_/*T*_F_, yet similar signal magnitudes. Clearly, the signal is broader for larger *T*_e_/*T*_F_, indicating faster thermal transport. We repeat these measurements for a range of *T*_e_ and *T*_F_ values and quantify the initial heat spreading using Gaussian functions, with widths *σ*_x_ and *σ*_y_, to describe ∆*I*_TE_ at Δ*t* = 0 as a function of Δ*x* or Δ*y* (Fig. 3e,f and Supplementary Fig. 5). As expected for a crossover from the diffusive Fermi-liquid regime to the hydrodynamic Dirac-fluid regime, both spatial spreads *σ*_x_ and *σ*_y_ increase substantially for increasing ratio *T*_e_/*T*_F_. These spreads correspond to a diffusivity up to 40,000 cm^2^ s^−1^ (Methods), similar to the 70,000 cm^2^ s^−1^ we found earlier.

We compare our experimental results to Boltzmann transport calculations following refs. ^6,18^, including carrier interactions and long-range impurity scattering. We model impurities as Thomas–Fermi screened Coulomb scatterers of density 0.24 × 10^12^ cm^−^^2^. Figure 3g shows the calculated thermal diffusivity *D* as a function of *T*_F_ and *T*_e_, when considering only the hydrodynamic term due to electron–electron interactions, relevant in the hydrodynamic window where |Δ*t*| < *τ*_mr_. A higher electron temperature, or lower Fermi temperature, leads to strongly increased diffusivity, signalling a crossover from the Fermi-liquid to the Dirac-fluid regime. This is the same qualitative trend as for the experimental data taken at Δ*t* = 0 in Fig. 3e–f, where a larger initial width originates from a larger diffusivity, thus supporting our interpretation of a hydrodynamic crossover.

A more quantitative comparison shows that the calculated *D* in the diffusive regime is around 2,000 cm^2^ s^−1^ (Fig. 3h), in quantitative agreement with the experiment in the diffusive regime. The obtained thermal diffusivity in the hydrodynamic window close to the Dirac point reaches values above 100,000 cm^2^ s^−1^, even higher than our experimental estimates of 35,000–70,000 cm^2^ s^−1^. Using the calculated diffusivities, we estimate the spatial spread at time zero *σ*_calc_ (Methods), as shown in Fig. 3g. These are similar to the experimentally obtained ones, thus confirming our conclusion of highly efficient heat spreading in the Dirac-fluid regime at room temperature, with a diffusivity that is almost two orders of magnitude larger than in the diffusive regime. We note that the theoretical calculations predict that even higher diffusivities are attainable.

Finally, we discuss the three-dimensional (3D) thermal conductivity, to assess the ability to transport useful amounts of heat. We find roughly 100 W m^−^^1^ K^−1^ in the diffusive regime (Methods), in agreement with ab initio calculations^29^. In the Dirac-fluid regime, with an electron temperature of roughly 1,000 K, we obtain a thermal conductivity of 18,000–40,000 W m^−^^1^ K^−1^. This is in agreement with ref. ^15^, where values up to 100,000 W m^−^^1^ K^−1^ were predicted theoretically for large *T*_e_/*T*_F_. The thermal conductivity we obtain is about three orders of magnitude larger than the one obtained in the Dirac-fluid regime at cryogenic temperatures^19^. Our results show that in the Dirac-fluid window the electronic contribution to heat transport can be much larger than the phononic contribution with a conductivity of >2,000 W m^−^^1^ K^−1^ (ref. ^30^), which is already exceptionally high and can also be enhanced hydrodynamically, as shown recently^31^. Thus, the Dirac electron-hole plasma can contribute strongly to thermal transport, extracting heat from hot spots much faster than predicted by classical limits.

In conclusion, our results show that the—until recently unreachable—physical phenomena associated with the Dirac fluid do not only offer an exciting playground for interesting physical phenomena, but also hold great promise for applications, for example in thermal management of nanoscale devices. We note that the quantum-critical behaviour can be switched on and off using a modest gate voltage and in systems prepared by standard fabrication techniques. Finally, we believe that the optoelectronic technique we have introduced—with the potential of increased spatial accuracy and temporal resolution—will be a valuable tool to reach a better understanding of the thermal behaviour of a broad range of quantum materials, with great promise for new technological applications.

## Methods

### Fabrication of split-gate thermoelectric device

The split-gate device with Hall geometry consists of exfoliated, single layer graphene encapsulated by hBN, prepared using standard exfoliation and dry transfer techniques. The hBN-graphene-hBN stack is placed on a predefined split-gate structure made of graphene, grown by chemical vapour deposition, where the gap between the two gates is roughly 100 nm, created via electron-beam lithography and reactive ion etching (RIE). The top hBN and graphene are etched into a Hall bar shape with laser lithography and RIE, keeping the split-gate intact and not etching completely through the bottom hBN. Finally, the Ti/Au side contacts are created by a further step of lithography, RIE and metal evaporation. The fabrication steps are shown in Supplementary Fig. 6.

### Spatiotemporal thermoelectric current microscopy setup

Our setup enables us to follow electronic heat spreading in space and time, because we use the thermoelectric signal generated by electronic heat interacting at a fixed location (the pn junction), while we vary the spatial displacement of our two laser pulses with respect to this junction and vary the temporal delay between the two ultrashort pulses. This means that we are following in space and time the diffusion of light-induced electronic heat from the location of light incidence to the pn junction. It is the thermoelectric effect at the pn junction, governed by the Seebeck coefficient, that generates our observable signal, the thermoelectric current. We note that although the value of the Seebeck coefficient itself changes when changing *E*_F_, and when entering the hydrodynamic regime^20^, this only affects the magnitude of the thermoelectric current, rather than how electronic heat is diffusing outside the pn junction, which is what we are following with our spatiotemporal technique.

A sketch of the setup is shown in Supplementary Fig. 7. A Ti:sapphire oscillator (886-nm centre wavelength, 76-MHz repetition rate), is split into two beam paths. Both beams are modulated with optical choppers, at frequencies *f*_1_ = 741 and *f*_2_ = 529 Hz. The relative time delay between the two pulses is controlled by a mechanical delay line. The spatial offset of one beam with respect to the other is controlled with a mirror galvanometer, while the position of the sample with respect to the beams is controlled with a piezo scanning stage. The beams are focused onto the sample with a ×40 0.6 numerical aperture objective lens. We collect the thermoelectric (TE) photocurrent between the source and drain contacts through the graphene sheet on either side of the junction via lock-in amplification. By demodulating the current signal at the difference frequency of the two modulation frequencies, *f*_2_ − *f*_1_ = 211.7 Hz, we isolate the signal caused by the interaction of both heating sources, which we call the interacting heat current ∆*I*_TE_. The temporal resolution of the setup of 200 fs is determined in the sample plane of the microscope (Supplementary Fig. 8). The spatial resolution defined by our spot sizes is below 1 μm, whereas the accuracy with which we can observe electronic heat spreading is determined by the signal-to-noise ratio, and is estimated to be below 100 nm.

### Estimating Fermi temperature controlled by gate voltage

During photocurrent measurements, the gate voltage *U*_x_ is applied to one side (x is ‘A’) or the other side (x is ‘B’) side of the split gate. We always apply a symmetric voltage around the experimentally determined Dirac point voltage $U_{{{\mathrm{x}}}}^{{{{\mathrm{DP}}}}}$: $U_{{{\mathrm{A}}}} = U_{{{\mathrm{A}}}}^{{{{\mathrm{DP}}}}} + {\Delta} U$ and $U_{{{\mathrm{B}}}} = U_{{{\mathrm{B}}}}^{{{{\mathrm{DP}}}}} - {\Delta} U$. The gate electrode and the graphene form a capacitor with the dielectric hexagonal boron nitride (hBN), with a thickness of *t*_hBN_ = 70 nm, and a relative permittivity of ${\it{\epsilon }}_{{{{\mathrm{hBN}}}}} = 3.56$. The carrier density *n* is calculated via $n = \frac{{{\it{\epsilon }}_0{\it{\epsilon }}_{{{{\mathrm{hBN}}}}}}}{{e\,t_{{{{\mathrm{hBN}}}}}}}{\Delta} U$, where $\epsilon_{0}$ is the vacuum permittivity. We calculate the Fermi energy *E*_F_ and the Fermi temperature *T*_F_ via $E_{{{\mathrm{F}}}}^2$ = *πħ*^2^$v_{{{\mathrm{F}}}}^2 \cdot n$ and $T_{{{\mathrm{F}}}} = \frac{{E_{{{\mathrm{F}}}}}}{{k_{{{\mathrm{B}}}}}},$ where *k*_B_ is the Boltzmann constant.

### Estimating carrier temperature controlled by laser power

The thermoelectric photovoltage is assumed to be proportional to the time-averaged increase of the electronic temperature *T*_e_ above the ambient temperature *T*_0_, as in ref. ^32^. The sublinear dependence of the thermoelectric current *I*_TE_ on optical power for the device under study here for illumination with a single pulsed laser (*λ* = 886 nm) is shown in Supplementary Fig. 9. With a linear temperature scaling of the electronic heat capacity for graphene away from the Dirac point, *C*_e_(*T*) = γ*T*, we integrate the heat energy per unit area d*Q* = *C*_e_d*T*, that is, $\mathop {\smallint }\limits_{Q_0}^{Q_0 + {\Delta} Q} {{{\mathrm{d}}}}Q = \mathop {\smallint }\limits_{T_0}^{T_e} \gamma T{{{\mathrm{d}}}}T$. With the incident power *P* proportional to the absorbed heat energy per unit area ∆*Q*, we find that the peak *T*_e_ as a function of the laser power *P* scales as in ref. ^32^, $T_e = \root {2} \of {{T_0^2 + bP}}$. Here, the parameter *b* is defined via *bP* = 2∆*Q/*γ, and is used to convert incident power to peak electron temperature (Supplementary Fig. 9).

### Simulation of the experiment

A detailed description of the simulation can be found in Supplementary Note 1 and Supplementary Fig. 10. In brief, we calculate the spatiotemporal evolution of electronic heat generated by the two optical pulses in the graphene sheet via the heat equation with a finite difference method. We define Gaussian heating pulses and calculate their temperature rise via the experimentally measured non-linear power scaling. We extract the differential TE current contribution as a function of *∆x* and *∆t* by the difference of the heating at the pn junction region in the presence of both pulses with respect to simulations with only one pulse at a time, analogous to the experimental difference-frequency demodulation.

### Quantifying the spatial spread

The following analysis is conducted both on the experimental and the simulated data of ∆*I*_TE_(∆*x*, ∆*t*) for ‘symmetric experiments’ with optical pulses incident at a distance ∆*x* on each side of the *pn-*junction (Figs. 1 and 2). For each ∆*t* of the datasets ∆*I*_TE_(∆*x*, ∆*t*) we calculate the width of the signal via the second moment, which for an ideal Gaussian profile is equal to the squared Gaussian width *σ*^2^. The second moment is calculated from the pixels ∆*x*_*i*_ (*i* = 1,…, *N*) via

$$\begin{array}{rcl}< {\Delta} x^2 > ({\Delta} t) & = & \frac{{\mathop {\sum }\nolimits_i \left| {{\Delta} x_i - \overline {{\Delta} x} } \right|^2{\Delta} I_{{{{\mathrm{TE}}}}}({\Delta} x_i,{\Delta} t)}}{{\mathop {\sum }\nolimits_i {\Delta} I_{{{{\mathrm{TE}}}}}({\Delta} x_i,\,{\Delta} t)}},\,{{{\mathrm{with}}}}\,{{{\mathrm{the}}}}\,{{{\mathrm{mean}}}} \\\overline {{\Delta} x} & = & \frac{{\mathop {\sum }\nolimits_i {\Delta} x_i{\Delta} I_{{{{\mathrm{TE}}}}}({\Delta} x_i,{\Delta} t)}}{{\mathop {\sum }\nolimits_i {\Delta} I_{{{{\mathrm{TE}}}}}(x_i,t)}}.\end{array}$$

We note that the minimum second moment at the focus <∆*x*^2^>_focus_ of 0.56 µm^2^ comes from simulating the symmetric experiment, using as input the measured Gaussian beam width at the focus σ_focus_^2^ = 0.14 µm^2^ (Supplementary Note 2). For the ‘asymmetric experiments’ with one optical pulse always incident on the pn junction (data for Fig. 3), we always consider the spatial profile only at time zero. Here we find that Gaussian fits with a background give the most reliable results. The entire set of data is shown in Supplementary Fig. 5. For each dataset ∆*I*_TE_(∆*x*) or ∆*I*_TE_(∆*y*) taken at ∆*t* = 0, we perform Gaussian fits using the function ${{{\mathrm{f}}}}({\Delta} x) = a\exp \left( { - \frac{{{\Delta} x^2}}{{2\sigma ^2}}} \right) + b$, where the Gaussian squared width *σ*^2^ indicates the thermal spreading. Here, the minimum simulated Gaussian widths are (*σ*_*x*_^2^)_focus_ = 0.34 µm^2^ and (*σ*_*y*_^2^)_focus_ = 0.44 µm^2^ (Supplementary Note 2). The experimentally obtained widths from this dataset as function of gate voltage and optical power are also shown in Supplementary Fig. 5, showing an increase with power, that is a larger *T*_e_, and an increase towards the Dirac point, that is a smaller *T*_F_. We estimate the theoretical Gaussian widths in Fig. 3g using $\sigma _{{{{\mathrm{calc}}}}}^2$ = (*σ*_*x*_^2^)_focus_ + 2*D* Δ*t*_IRF_, where *D* are the calculated diffusivities.

### Electrical measurements

We characterize our device electrically with four-probe measurements (Extended Data Fig. 1), finding a charge mobility *μ* of 30,000–50,000 cm^2^ Vs^−1^, depending on carrier density. The measured mobilities correspond to a momentum relaxation time 𝜏_mr_ of 300–500 fs. These relaxation times are longer than the temporal resolution (the IRF) of our measurement technique, Δ*t*_IRF_ ≅ 200 fs, thus allowing us to probe our system before and after momentum relaxation occurs, that is in the non-diffusive and diffusive regime. We use these measured charge mobilities to calculate the expected thermal diffusivity via the Einstein relation^33,34^ $\mu _{{{{\mathrm{e}}}}/{{{\mathrm{h}}}}} = \frac{e}{{n_{{{{\mathrm{e}}}}/{{{\mathrm{h}}}}}}}\frac{{\partial n_{{{{\mathrm{e}}}}/{{{\mathrm{h}}}}}}}{{\partial E_{{{\mathrm{F}}}}}}D_{{{{\mathrm{e}}}}/{{{\mathrm{h}}}}}$, where *e* is the elementary charge, *E*_F_ is the Fermi energy and *n*_e/h_ is the electron/hole carrier density. For highly doped graphene ($E_{{{\mathrm{F}}}} \gg k_{{{\mathrm{B}}}}T$) the carrier density expression $n_{{{{\mathrm{e}}}}/{{{\mathrm{h}}}}} = \frac{{E_{{{\mathrm{F}}}}^2}}{{\pi \hbar ^2v_{{{\mathrm{F}}}}^2}}$, leads to the simple relation:$D_{{{{\mathrm{e}}}}/{{{\mathrm{h}}}}} = \frac{{E_{{{\mathrm{F}}}}}}{{2e}}\mu _{{{{\mathrm{e}}}}/{{{\mathrm{h}}}}}$. We note that we obtain the identical result by calculating *D* from the ratio of the 2D thermal conductivity *κ*_e,2D_ and the electronic heat capacity *C*_e_ and using the Wiedemann–Franz law: $\kappa _{{{{\mathrm{e}}}},2{{{\mathrm{D}}}}}/\sigma = \pi ^2/3\cdot (k_{{{\mathrm{B}}}}/e)^2T_{{{\mathrm{e}}}}$, where *k*_B_ is the Boltzmann constant and *e* the elementary charge, together with the conductivity *σ* = *neμ* and the following heat capacity for graphene (valid for *T*_e_ < *T*_F_): $C_{{{\mathrm{e}}}} = \frac{{2\pi \varepsilon _{{{\mathrm{F}}}}k_{{{\mathrm{B}}}}^2T_{{{\mathrm{e}}}}}}{{3\hbar ^2v_{{{\mathrm{F}}}}^2}}$. Given the measured mobilities, we expect thermal diffusivities around 2,000 cm^2^ s^−1^ for our sample.

### Thermal diffusivity and conductivity of the Dirac fluid

We estimate the enhanced thermal diffusivity of the Dirac fluid by comparing the measured width at time zero <Δ*x*^2^>_min_ to the expected width <Δ*x*^2^>_focus_ explained above, via *D* = (<Δ*x*^2^>_min_ − <Δ*x*^2^>_focus_)/2Δ*t*_IRF_. We find values up to 74,000 cm^2^ s^−1^ for the symmetric scan (Fig. 2), and 29,000 and 39,000 cm^2^ s^−1^ for the *x* and *y* directions of the asymmetric scan (Fig. 3), respectively, where <Δ*x*^2^> is replaced with (*σ*_*x*_^2^) and (*σ*_*y*_^2^), respectively. The same calculation for a second device (Supplementary Note 3 and Extended Data Fig. 2) gives a diffusivity of 100,000 cm^2^ s^−1^. The 3D thermal conductivity *κ*_3D_ of the Dirac fluid is calculated from the diffusivity *D* and the electronic heat capacity *C*_e_, via *κ*_3D_ = *DC*_e_/*d*, where *d* is the thickness of graphene, 0.3 nm. For the Dirac fluid, we have *T*_e_ > *T*_F_, and therefore use the ‘undoped’ electronic heat capacity^35^ $\frac{{18\,\zeta (3)}}{{\pi (\hbar v_{{{\mathrm{F}}}})^2}}k_{{{\mathrm{B}}}}^3T_{{{\mathrm{e}}}}^2$, where $\zeta \left( 3 \right) \approx 1.202$. With the above estimate *D* = 35,000–70,000 cm^2^ s^−1^ and *T*_e_ = 1,000 K, we obtain the 3D thermal conductivity *κ*_3D_ = 18,000–40,000 W mK^−1^. This corresponds to a 2D *κ*_2D_ > 5 μW K^−1^. This value is orders of magnitude larger than the value found in ref. ^19^. The reason for this is that our electron temperature is more than ten times higher, and therefore the electronic heat capacity is >100× higher. Furthermore, we reach a *T*_e_/*T*_F_ > 3, while their maximum *T*_e_/*T*_F_ was around two, which means that we are further in the Dirac-fluid regime with its diverging thermal diffusivity.

### Dirac-fluid crossover temperature

Following the treatment in ref. ^14^, we find the crossover temperature from Fermi liquid to Dirac fluid, as a function of Fermi temperature as

$$T_{{{{\mathrm{cross}}}}}(T_{{{\mathrm{F}}}}) = T_{{{\mathrm{F}}}}\left( {1 + \lambda \ln \left( {\frac{{T_0}}{{T_{{{\mathrm{F}}}}}}} \right)} \right),$$

where $\lambda = {{{\mathrm{e}}}}^2/16\epsilon _0{\it{\epsilon }}_{{{\mathrm{r}}}}v_{{{\mathrm{F}}}}\hbar \approx 0.55/{\it{\epsilon }}_{{{\mathrm{r}}}}$ for graphene with the dielectric environment ${\it{\epsilon }}_{{{\mathrm{r}}}} \approx 3.56$ for hBN. The temperature $T_0 = \frac{{2\hbar v_{{{\mathrm{F}}}}\sqrt \pi }}{{3^{3/4}k_{{{\mathrm{B}}}}a_0}} \approx 8.4\times 10^4\,{{{\mathrm{K}}}}$, with the inter-atomic distance $a_0 = 1.42\times 10^{ - 10}\,{{{\mathrm{m}}}}$. The resulting crossover temperature is shown in Fig. 1b. We note that the relatively high refractive index of the hBN encapsulant makes the Dirac fluid more easily accessible, as it lowers the crossover temperature compared to vacuum, by a factor of about two for the range of *T*_F_ values studied here.

## Online content

Any methods, additional references, Nature Research reporting summaries, source data, extended data, supplementary information, acknowledgements, peer review information; details of author contributions and competing interests; and statements of data and code availability are available at 10.1038/s41565-021-00957-6.

## Supplementary information

Supplementary Information Supplementary Notes 1–4 and Figs. 1–10.

## Data Availability

The data that support the findings of this study are available from the corresponding author on reasonable request.

## Extended data

are available for this paper at 10.1038/s41565-021-00957-6.
