# Supplementary material for: Observation of giant and tunable thermal diffusivity of a Dirac fluid at room temperature
Source: Nat Nanotechnol. 2021 Aug 23;16(11):1195–200. doi: 10.1038/s41565-021-00957-6 (PMC8592840; doi:10.1038/s41565-021-00957-6)
Supplement: Supplementary file 1 — Supplementary Notes 1–4 and Figs. 1–10. [file 41565_2021_957_MOESM1_ESM.pdf]

nature portfolio

https://doi.org/10.1038/s41565-021-00957-6

Supplementary information

Observation of giant and tunable thermal diffusivity of a Dirac fluid at room temperature

In the format provided by the authors and unedited

Supplementary information for:

Observation of giant and tuneable thermal diffusivity of Dirac fluid at room temperature

Alexander Block[1,2], Alessandro Principi[3], Niels C.H. Hesp[1], Aron W. Cummings[2], Matz Liebel[1], Kenji Watanabe[4], Takashi Taniguchi[5], Stephan Roche[2,6], Frank H. L. Koppens[1,6], Niek F. van Hulst[1,6], Klaas-Jan Tielrooij[2,*]

[1]ICFO – Institut de Ciències Fotòniques, The Barcelona Institute of Science and Technology, Castelldefels, Spain
[2]Catalan Institute of Nanoscience and Nanotechnology (ICN2), BIST & CSIC, Campus UAB, Bellaterra, Spain
[3]School of Physics and Astronomy, University of Manchester, Manchester, UK
[4]Research Center for Functional Materials, National Institute for Materials Science, 1-1 Namiki, Tsukuba 305-0044, Japan
[5]International Center for Materials Nanoarchitectonics, National Institute for Materials Science, 1-1 Namiki, Tsukuba 305-0044, Japan
[6]ICREA, Institució Catalana de Recerca i Estudis Avançats, Barcelona, Spain
*Correspondence to: klaas.tielrooij@icn2.cat

Contents:

Supplementary Note 1: Simulation of the experiment
Supplementary Note 2: Broadening at time zero
Supplementary Note 3: Second device
Supplementary Note 4: Third device

Suppl. Fig. 1: Scanning-edge beam profiling
Suppl. Fig. 2: Device imaging
Suppl. Fig. 3: Comparison between second moment analysis and Gaussian fitting
Suppl. Fig. 4: Focusing to ensure minimum spot size for ΔI_{TE} measurements
Suppl. Fig. 5: Raw data for Fig. 3e-f
Suppl. Fig. 6: Hall-bar split gate device fabrication
Suppl. Fig. 7: Schematic of experimental setup
Suppl. Fig. 8: Temporal resolution
Suppl. Fig. 9: Extraction of electron temperature
Suppl. Fig. 10: Spatiotemporal heat simulation

Supplementary Note 1: Simulation of the experiment

Experimentally, we have used two distinct measurement configurations: for the data presented in Fig. 2, the two laser pulses are spatially offset symmetrically with respect to the gate junction region (by $\Delta x/2$ from the junction) by synchronized movement of the galvo mirrors (by Δx) and the piezo sample stage (by $\Delta x/2$); for the data presented in Fig. 3, where we focus on the hydrodynamic time window, we use a simpler "asymmetric" measurement geometry that gives a larger signal. Here, we keep one beam fixed on the junction while scanning the other beam by Δx, across the junction (Fig. 3e), and by Δy, along the junction (Fig. 3f), with fixed sample stage, while moving the galvo mirrors only.

We simulate the "symmetric" experiment of Fig. 1-2 by letting two laser pulses impinge on graphene on either side of the *pn*-junction. Upon absorption, the electron temperature rises with peak amplitude ΔT_e with respect to the non-excited region. The simulation space is explained schematically in Suppl. Fig. 10.

The absorbed heat ΔQ_α (α = 1 or 2) due to the absorption of each pulse, respectively, is modeled as a Gaussian in space ($\boldsymbol{x} = (x, y)$) and a delta-pulse in time (t) (with a time grid resolution on the order of the pulse width),

$$\Delta \boldsymbol{Q}_{\boldsymbol{\alpha}}(\boldsymbol{x}, \boldsymbol{t}) = \boldsymbol{P}_{\boldsymbol{\alpha}} \exp\left(-\frac{(\boldsymbol{x}-\Delta \boldsymbol{x}_{\boldsymbol{\alpha}})^2 + (\boldsymbol{y}-\Delta \boldsymbol{y}_{\boldsymbol{\alpha}})^2}{2\boldsymbol{\sigma}_{\mathbf{focus}}^2}\right) \boldsymbol{\delta}(\boldsymbol{t} - \Delta \boldsymbol{t}_{\boldsymbol{\alpha}}) \,. \qquad \text{(S1)}$$

Here, P_α is the amplitude, Δx_α and Δy_α are the spatial offsets from the center of the junction across and along the junction axis, respectively, Δt_α is the pulse delay, and σ_{focus} is the laser focal width. Note that t and x represent the "lab"-coordinates, which will be summed over in the calculation of the photocurrent (Eq. S4), while the variables Δt and Δx will be used, respectively, as the pulse delay and spatial offsets of the beams as they are scanned. In particular, we set $\Delta t_1 = 0$, $\Delta t_2 = \Delta t$; and, depending on the scanning mode, $\Delta x \equiv \Delta x_1 - \Delta x_2 = 2\Delta x_1$ for the symmetric scan (data of Fig. 2), or $\Delta x_1 = 0$, $\Delta x_2 = \Delta x$ for the asymmetric scan (data of Fig. 3).

We calculate the elevated electron temperature ΔT_e from the absorbed heat energy ΔQ, according to a nonlinear function $f_{nonlinear}$ known from the power dependence of the electron temperature[1]. Here, in contrast to previous work, we explicitly include both the spatial and temporal dependence of this process,

$$\Delta T_e(\boldsymbol{x}, t) = f_{\text{nonlinear}}\big(\Delta Q(\boldsymbol{x}, t)\big) = \sqrt[2]{T_0^2 + b\,\Delta Q} - T_0 \,, \qquad \text{(S2)}$$

where $T_0 = 293$ K is the ambient temperature. As the parameter $b = 1.7 \cdot 10^5$ K^2/µW is determined experimentally (see Suppl. Fig. 9), the heating amplitude P_α is inserted as the time averaged laser power at the sample, i.e. in units of µW. Hence, there is no need to correct for the absorbed power fraction or optical losses.

The total temperature rise $\Delta T_{\mathrm{e}}^{\mathrm{tot}}$ depends on the absorbed heat of both pulses and is calculated as

$$\Delta T_{\mathrm{e}}^{\mathrm{tot}}(\boldsymbol{x},t) = f_{\mathrm{nonlinear}}(\Delta Q_1(\boldsymbol{x},t) + \Delta Q_2(\boldsymbol{x},t)).$$

Next, we let the heat evolve according to a heat equation with diffusivity D, and an additional decay term that accounts for cooling of the hot-electron system, where we use a time constant of τ_{cool} = 2 ps [1],

$$\frac{\partial T(\boldsymbol{x},t)}{\partial t} = \nabla\big(D\nabla T(\boldsymbol{x},t)\big) - \tau_{\mathrm{cool}}^{-1} T(\boldsymbol{x},t)\ . \quad \text{(S3)}$$

We calculate this evolution with a finite difference method (Crank-Nicolson) on a regular, rectangular grid. Suppl. Fig. 10 shows the heat evolution in the "lab"-coordinate space (x, t) for a particular set of offsets (Δx, Δt).

The two independently electrostatically gated regions of the split gate sample are characterized by different Seebeck coefficients, S_1 and S_2, where $S_1 = -S_2$, since we always use symmetric gating. We use one value for the diffusivity, i.e. equating the hole and electron diffusivity. A local voltage U_{TE} is created due to the photothermoelectric effect. The voltage reads[2]

$$U_{\mathrm{TE}} = (S_2 - S_1)\ \Delta T_{\mathrm{e}}\ .$$

Finally, we note that the photocurrent I_{TE} comes from the time-averaged increase in electron temperature ΔT_{e} at the location of the split-gate, as this is the location of the stepwise difference in Seebeck coefficients where the photovoltage is created. The following sum is therefore taken over the junction region in space, and over the full simulation time,

$$I_{\mathrm{TE}} = \mathrm{A}\ \sum_t \sum_{\boldsymbol{x}_{\mathrm{junction}}} \Delta T_e(\boldsymbol{x},t), \quad \text{(S4)}$$

where the photocurrent $I_{\mathrm{TE}} = U_{\mathrm{TE}}/R$ is given by the local photovoltage and the total device resistance R. The proportionality constant A relates temperature and current, i.e., includes the Seebeck coefficient step ($S_2 - S_1$) and R. In our simulation, we set A = 1 and calculate the photocurrent in arbitrary units.

We isolate the decrease in photocurrent due to the interaction of the heat of the two modulated sources, Q_1 and Q_2. This is analogous to the experimental demodulation at the difference frequency with the double-chopper technique, as introduced in the main text. Here, we define the differential thermoelectric current ΔI_{TE} as

$$\Delta I_{\mathrm{TE}} = I_{\mathrm{TE}}(\Delta Q_1) + I_{\mathrm{TE}}(\Delta Q_2) - I_{\mathrm{TE}}(\Delta Q_1 + \Delta Q_2), \quad \text{(S5)}$$

where the individually calculated currents are obtained by simulating the experiment, with only one ($I_{\mathrm{TE}}(\Delta Q_1)$), the other ($I_{\mathrm{TE}}(\Delta Q_2)$), and both heating pulses ($I_{\mathrm{TE}}(\Delta Q_1 + \Delta Q_2)$). We then

separately calculate the heat evolution (Eq. S3), and TE current evaluation (Eq. S4) for all 3 cases to arrive at ΔI_{TE} (via Eq. S5).

This is the procedure for one given combination of Δx and Δt. We then repeat the procedure for a range of values for Δx and Δt, in order to obtain the differential thermoelectric photocurrent ΔI_{TE} as a function of Δx and Δt.

The simulations of the "asymmetric" experiments of Fig. 2 proceed along the same line, with different initial conditions for the two laser pulses.

Supplementary Note 2: Broadening at time zero

In this Supplementary Note we discuss possible experimental and physical effects that could lead to the observed broadening around zero time delay, which we attribute to hydrodynamic heat diffusion.

Second moment analysis

As described in Methods, we use a second moment analysis to quantify the spatial extent of our ΔI_{TE} (Δx) profiles for the symmetric scanning and Gaussian fitting for asymmetric scanning. Here, we compare the two methods. For perfectly Gaussian distributions, with no offset or noise, the two are equivalent and lead to the same result $\langle \Delta x^2 \rangle = \sigma_{Gauss}^2$. However, in the case of noisy signals, with offsets, or non-Gaussian distributions this is not necessarily the case[3].

Suppl. Fig. 3 shows a comparison between the two analysis methods applied to the data of Fig. 2 of the manuscript. We observe a difference in the time zero width of 30-40 %, while the gate dependent trend is the same for both analysis methods. For example, for the gate voltage of 4.5 V (E_F = 130 meV), the time zero width is 2.5 µm^2 for second moment analysis, while it is (1.8 ± 0.2) µm^2 for the Gaussian fitting. The errorbars show the 68% confidence intervals, while the second moment analysis does not produce an error.

While the second moment possibly overestimates the width somewhat, the time-zero width is still significantly larger than the 0.56 µm^2 expected from purely diffusive transport. Hence, a fast (super-diffusive) transport during the first few hundred fs is required to explain the observed data.

Experimental focusing conditions

We have taken special care to the focusing conditions. We have characterized our beams with a scanning edge technique, spatial scans, and performed z-dependent TE current measurements. Suppl. Fig. 2 shows a spatial photocurrent scan, with spatial resolution of around σ_{focus}^2 = 0.09 µm^2, consistent with scanning edge measurements, showing σ_{focus}^2 = 0.14 µm^2 (shown in Suppl. Fig. 1).

Note that the width σ_{focus}^2 acts as an *input* to the simulations (c.f. Eq. S1), while the *output* $\langle \Delta x^2 \rangle_{focus}$ comes from the full simulation run, for Δt = 0 and as a function of Δx, and analyzed in terms of width along this Δx axis, after integration along the split-gate region and all of "lab"-time t (c.f. Eqs. S4-5). For symmetric measurements as shown in Fig. 2, the minimum output width (second moment analysis) obtained using the input σ_{focus}^2 = 0.14 µm^2 is $\langle \Delta x^2 \rangle_{focus}$ = 0.56 µm^2. For asymmetric measurements as shown in Fig. 3, the minimum output width (Gaussian fit) is $(\sigma_x^2)_{focus}$ = (0.34 – 0.40) µm^2 and $(\sigma_y^2)_{focus}$ = (0.44 – 0.53) µm^2, for the optical power range of (4 - 20) µW.

Suppl. Fig. 4a shows ΔI_{TE} maps, taken at Δt = 0, as a function of beam offset (Δx, Δy), as well as sample height (z-axis, along the beam axis). A clear minimum in the spatial extent can be observed. We extract line profiles for the two scanning dimensions (Suppl. Fig. 4b) and calculate

the signal width σ_α^2 for both dimensions (α = x, y) as extracted from Gaussian fits at each z-position (equivalent to second moment $\sigma_\alpha^2 = < \Delta\alpha^2 >$), shown in Suppl. Fig. 4c.

The high spatial resolution achieved, together with the optimal focusing conditions established, we exclude the possibility of extracting artificially high spatial extents due to weak focusing.

Considerations of signal-to-noise ratio

Suppl. Fig. 5c shows the absolute signal strength for the data analyzed in Fig. 3e-f of the manuscript. The signal strength does not correlate with the changes in extracted width, hence excluding a broadening artifact related to signal-to-noise ratio. In fact, the powers and gate voltages of Fig. 3a-d of the manuscript show ΔI_{TE} maps with very similar signal strength, but clear differences in the spatial extent.

Ballistic motion via Monte Carlo simulations

To estimate the effect of ballistic motion on the broadening of an initially localized distribution of hot carriers, we first perform Monte Carlo simulations. To start, we choose N =10^7 point particles starting from a random (x, y) position, distributed within a Gaussian with width σ_{focus}^2 = 0.14 µm^2, simulating the laser focus which we extracted experimentally with a scanning edge technique. Then, we let each particle move on a straight trajectory into a random direction for a distance d = v_F t. We create a histogram of the particles' final spatial distribution at time t and fit the distribution with a Gaussian function. Extended Data Fig. 4 shows the final distribution's width as a function of t for different values of the Fermi velocity. For the standard value of 1.0 µm/ps, we find that ballistic spreading within 250 fs leads to a final width of σ_{ball}^2 = 0.18 µm^2. When inserting this value to the simulation, we get a minimum $<\Delta x^2>_{ball}$ = 0.68 µm^2, as shown in Fig. 2e of the manuscript. Hence, this effect is not enough to explain the broadening we observe of $<\Delta x^2>_{min}$ > 2 µm^2. Furthermore, even if we assume that low carrier densities the Fermi velocity might take values up to 1.4 µm/ps, we get only a 10% increase in the final width at 250 fs, well below our observed effect.

Ballistic motion via Quantum simulation

In the quantum mechanical simulations of ballistic transport, we first define the single-electron wave function as a Gaussian wave packet at $t = 0$, $\psi(\vec{r},0) = \frac{1}{\sqrt{2\pi}\sigma_r}\exp\left(-\frac{|\vec{r}-\vec{r}_0|^2}{4\sigma_r^2}\right)\exp\left(-i\vec{k}_0\cdot\vec{r}\right)$, where σ_r is the position uncertainty of the electron, and $\left(\vec{r}_0,\vec{k}_0\right)$ defines its central position in real space and momentum space. The corresponding uncertainty in momentum space is $\sigma_k = 1/(2\sigma_r)$. We then evolve this wave function in time via $\psi(\vec{r},t) = \hat{U}(t)\psi(\vec{r},0)$, where $\hat{U}(t) = \exp\left(-i\hat{H}t/\hbar\right)$ is the time evolution operator and $\hat{H}$ is the real-space tight-binding Hamiltonian of graphene with nearest-neighbor hopping integral $\gamma_0 = 3$ eV. The top panels of Extended Data Fig. 4a show the time evolution of the single-electron density $|\psi(\vec{r},t)|^2$, where we have chosen $\sigma_r = 3.3$ nm and $\vec{k}_0 = K$ such that the wave function is centered around the graphene Dirac point in momentum space. This single-electron wave function spreads outward from its initial distribution at a velocity equal to the Fermi velocity of graphene, $v_F = 3/2 \cdot \gamma_0 a_{cc}/\hbar = 1$ µm/ps, where $a_{cc} = 0.142$ nm is the nearest-neighbor carbon distance.

To model the spread of an ensemble of independent electrons, we evaluate the total electron density $n(\vec{r},t) = |\psi(\vec{r},t)|^2 * g(\vec{r})$, where $g(\vec{r})$ is the initial distribution of the electron ensemble and '$*$' is the convolution operation. The bottom panels of Extended Data Fig. 4a show the evolution of this ensemble electron density assuming $g(\vec{r})$ is a Gaussian distribution with a width $\sigma = 38$ nm. Note that in these panels, for visualization purposes we have multiplied the time and length scales by a factor of 10 to match the experimental scales. In Extended Data Fig. 4b we show the width of the ensemble electron density as a function of time, starting from the laser focus width of $\sigma^2_{\mathrm{focus}} = (0.38\ \mu\mathrm{m})^2 = 0.14\ \mu\mathrm{m}^2$.

The quantum simulation matches well with the classical simulation, and both predict a width that is much smaller than what is measured in the experiments at $t = 250$ fs.

Discussion

We have presented various mechanisms that could lead to a larger-than-expected time-zero width, such as an experimental artifact due to non-optimal focusing, a mathematical artifact due to the second moment analysis, and ballistic motion. While the focusing conditions have been controlled and quantified, neither the effects of ballistic motion (10%), nor the second moment analysis (30-40 %), nor a combination of these effects, could lead to the observed broadening of the squared width of about 350%. This leads us to conclude that only ultrafast initial heat spreading (due to hydrodynamics) can explain our observations.

Supplementary Note 3: Second device

We used a second split-gate device that was prepared using similar fabrication methods, with the main differences that the split-gate geometry is composed of gold, rather than CVD graphene, and the graphene is not patterned into a Hall geometry. Further fabrication details for this sample are described in Ref. [4]. The mobility was found to be very similar to the device with Hall bar geometry: ~30,000 cm^2/Vs, thus allowing for the observation of non-diffusive regimes at ultrashort time scales.

Experimental results for the second device are shown in Extended Data Fig. 2. Here, the two illuminating heating pulses have wavelengths of 433 nm and 866 nm. The blue beam is fixed at the junction region, while the NIR beam is scanned (asymmetric scanning mode). Extended Data Fig. 2a shows spatiotemporal ΔI_{TE} datasets for three different gate voltages, ΔU = (0.4 - 0.8) V, corresponding to E_F = (46 - 65) meV in this device. The spatial extent as a function of Δt is shown in Extended Data Fig. 2b. A larger initial width for lower Fermi level (with fixed laser power) is observed, consistent with the results shown in Fig. 3e-f of the manuscript.

Extended Data Fig. 2c-e shows the time zero ΔI_{TE} focal plane scans for this device, as explained for the main (Hall-bar) device in the Suppl. Note 2 and Suppl. Fig. 4. The minimum width is significantly above the expected width $(\sigma_x^2)_{focus}$ = (0.30 – 0.35) μm^2 and $(\sigma_y^2)_{focus}$ = (0.33 – 0.40) μm^2, simulated from a purely diffusive process with initial beam sizes, $\sigma_{focus,886nm}^2$ = 0.14 μm^2 and $\sigma_{focus,443nm}^2$ = 0.07 μm^2, which were determined by a scanning edge technique. The ranges come from the dependence of optical heating pulse power in the experimental ranges of (4-20) µW. As before, the observation of minimum widths well above $(\sigma_{x/y}^2)_{focus}$, are attributed to super-diffusive transport in the hydrodynamic regime. For this device, we estimate a diffusivity in the Dirac-fluid regime of (4.5 μm^2 – 0.56 μm^2)/(2·200 fs) ≈ 100,000 cm^2/s, in agreement with the results obtained with the device discussed in the main text.

Supplementary Note 4: Third device

In order to provide additional evidence that supports our conclusion that the observed large heat spreading around time zero occurs due to hydrodynamic heat flow, we have performed a control experiment using a graphene sample with a lower mobility. For this we used a sample where – compared to the first and second device – the bottom hBN is replaced by SiO_2. This results in a lower mobility, while still giving a reasonable photocurrent. The results are shown in Extended Data Fig. 3.

We show a microscope image of the control sample in Extended Data Fig. 3a and electrical measurements in Extended Data Fig. 3b. We find a mobility around 8,500 cm^2/Vs, which is about 4x lower than the other two (hBN-encapsulated) devices. This means that the momentum relaxation time is below 100 fs, compared to around 350 fs for the other two devices. The hydrodynamic window is therefore significantly shorter, and also shorter than our instrument response function of 200 fs. Similar to the results in Figs. 3a-d of our manuscript, we measure the time-zero spatial maps while changing laser power (controlling T_e) and gate voltage (controlling T_F), see Extended Data Fig. 3c-d. We note that the range for T_F is limited for the third device, because the signal is too weak close to the Dirac point (due to charge puddles), and there is a large risk of SiO_2 starting to leak at higher gate voltages. We adjust the incident power such that the range of electron temperatures T_e is similar for the first (high-mobility) device and the third (low-mobility) device. We achieve this by making sure that the (one-laser) photovoltage is the same at similar doping level. Assuming that the Seebeck coefficients, cooling times and device geometries are similar, also the electron temperature will be similar in both devices. We find a photovoltage for both devices of nearly 40 µV for 60 µW on the first device and 666 µW on the third device. The larger power is probably needed because there is less absorption in the third device due to an interference effect in the oxide layer. We note that the electron temperature in the third device is likely larger as the geometry of the device is less favorable due to its longer *pn*-junction.

We compare the time-zero spatial width for different laser powers and gate voltages (see Extended Data Fig. 3e-f) for the first and third device. We quantify the time-zero spatial spread in the same way as we did for the original (first) device, and clearly, the width is systematically smaller for the third device with lower mobility and therefore shorter hydrodynamic time window (<100 fs). This shows that a smaller hydrodynamic window (due to faster momentum relaxation) gives rise to less spatial broadening around time zero, in agreement with our conclusion that the ultrafast broadening is the result of unconventional hydrodynamic heat spreading.

Supplementary Figure 1.

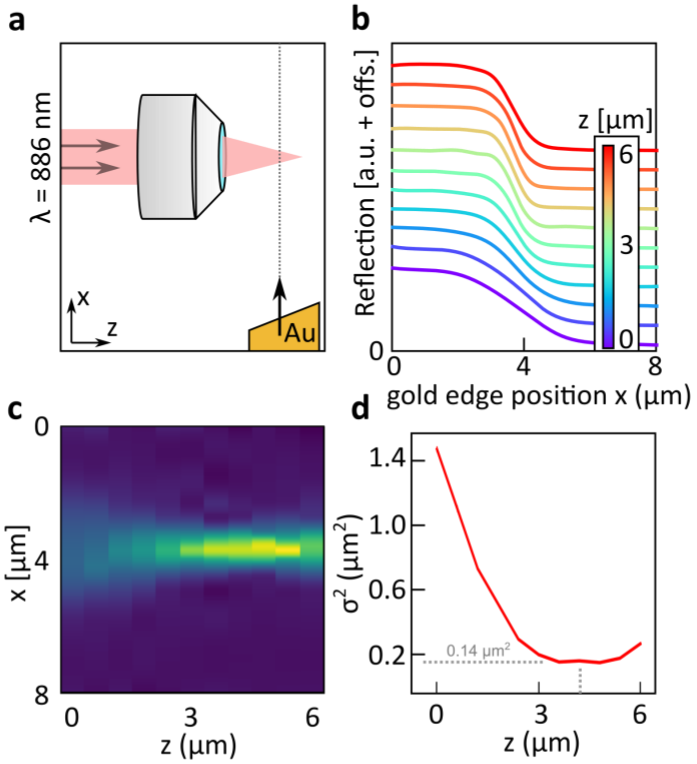

Suppl. Fig. 1: Scanning-edge beam profiling. (**a**) A 50 nm thin gold film with a sharp edge is placed in the sample plane and scanned through the beam in the *x*-direction for different sample heights *z*. (**b**) Reflected power is recorded as a function of position *x* of the gold edge. (**c**) The spatial derivative of the profiles along the *x*-axis represent the beam profile as a function of scanning direction, *x*, and axial offset, *z*. (**d**) Extracted width (σ^2) by Gaussian fits to line profiles, i.e., vertical cuts of **c**, showing the z-dependence. The minimum width, corresponding to the focus, was measured as σ_{focus}^2 = 0.14 μm^2. The same characterization was also done for the other spatial dimension, y, yielding similar results.

Supplementary Figure 2.

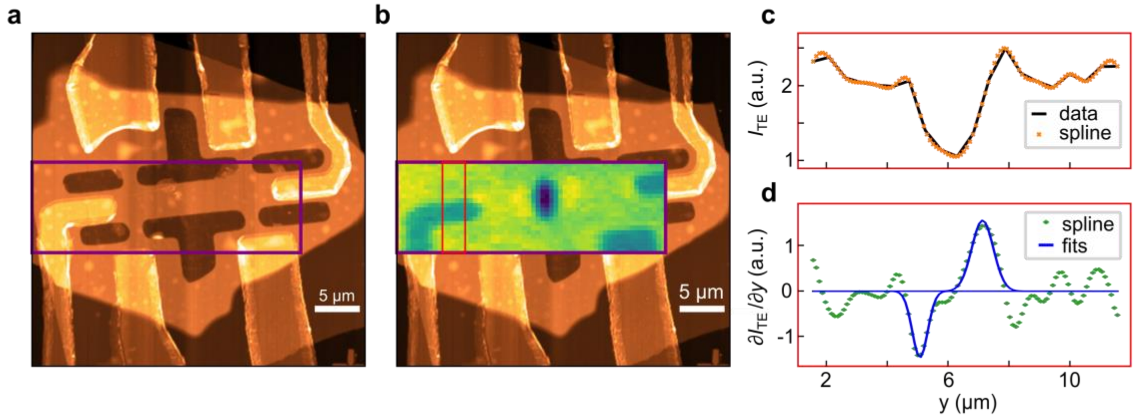

Suppl. Fig. 2: Device imaging. (a, b) AFM image of the device (adapted from Ref. [5]), with superimposed single laser photocurrent image (**b**). (**c**) Extracted spatial profile from the area of the red rectangle in B. (**d**) estimation of the point-spread function by extraction of the derivative and subsequent Gaussian fitting at the step regions. The resulting average width $\sigma_{PSF}^2 = 0.09\ \mu m^2$ is consistent with knife edge and optical characterization of the beams.

Supplementary Figure 3.

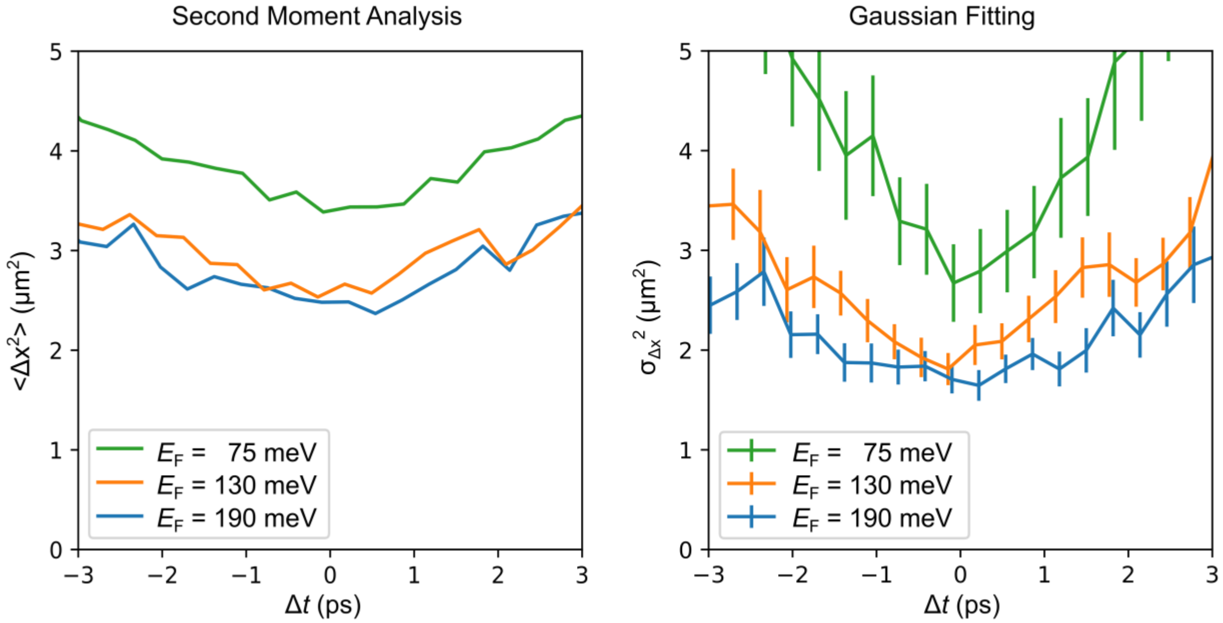

Suppl. Fig. 3: Comparison between second moment analysis and Gaussian fitting. The spatial extent of $\Delta I_{TE}(\Delta x, \Delta t)$ datasets are measured via the second moment (left) and Gaussian fitting (right). Both techniques show the same trends for the three different gating conditions. The second moment analysis produces a somewhat larger number, as is known to happen in the presence of noise[3]. However, the trend with increasing time delay is more reliable for the second moment analysis, as the spatial shape of the signal becomes increasingly less Gaussian.

Supplementary Figure 4.

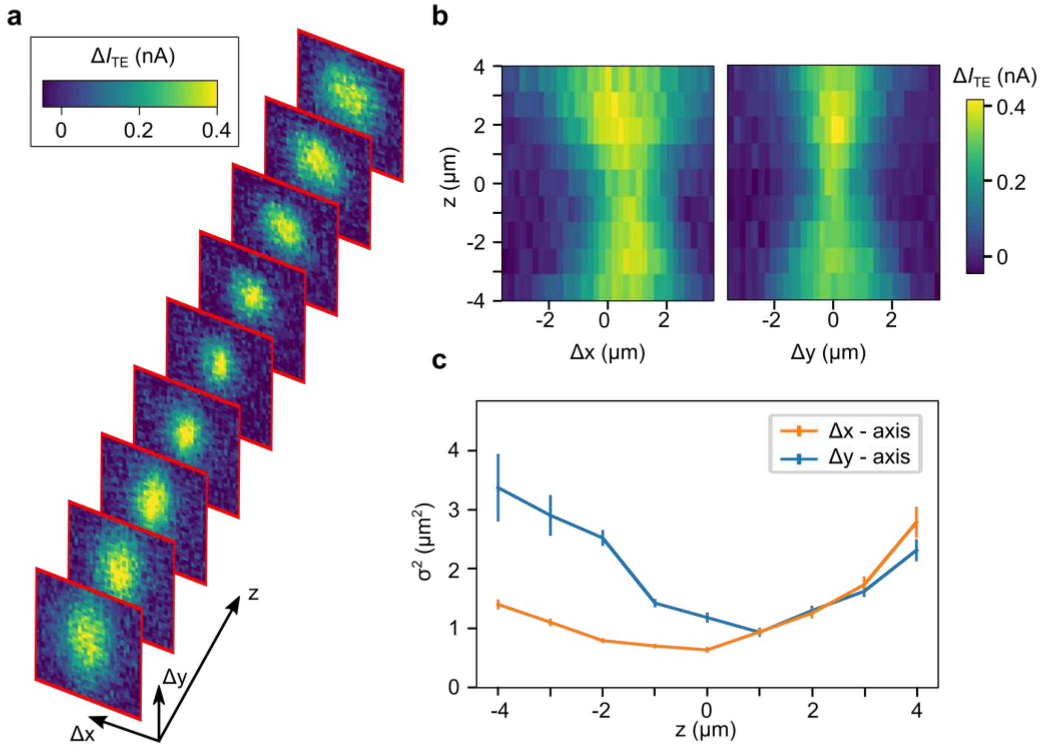

Suppl. Fig. 4: Focusing to ensure minimum spot size for ΔI_{TE} measurements. (**a**) ΔI_{TE} maps, taken at Δt = 0, as a function of beam offset (Δx, Δy), as well as sample height (z). (**b**) extracted line profiles for the two dimensions. (**c**) Resulting signal width σ^2 for both dimensions as extracted from Gaussian fits at each z-position.

Supplementary Figure 5.

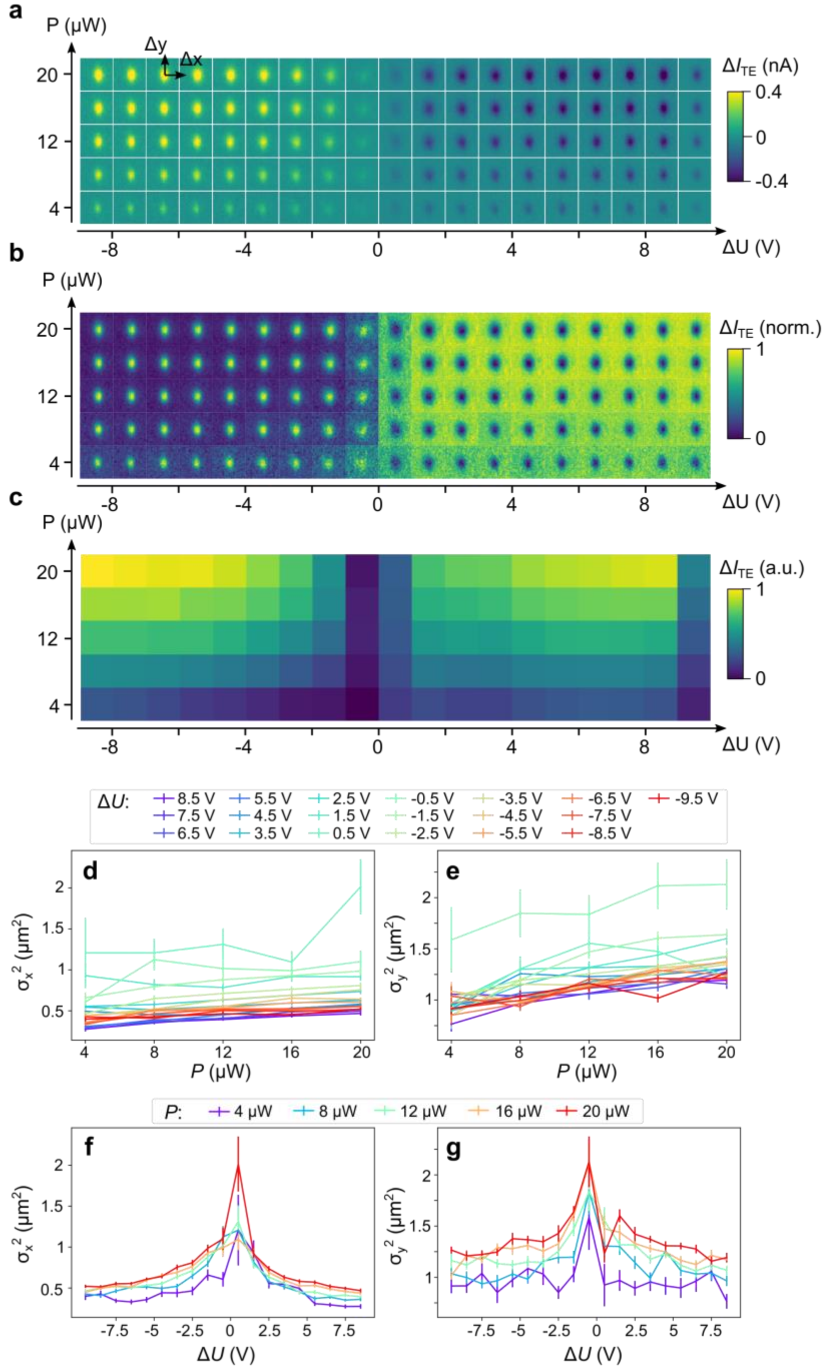

Suppl. Fig. 5: Raw data for Fig. 3e-f. (**a**) ΔI_{TE} maps, taken at $\Delta t = 0$, as a function of beam offset (Δx, Δy), for varying laser power and gate voltage. (**b**) Same as **a**, normalized for each map. (**c**) Signal strength (absolute) for each map. The lower signal strength does not correlate with the increase in width (c.f. Fig. 3e-f of the manuscript), hence excluding a broadening artifact due to worse signal-to-noise. (**d**-**f**) Vertical and horizontal cuts through Fig. 3e-f, illustrating the trends with power (T_e) and gate voltage (T_F).

Supplementary Figure 6.

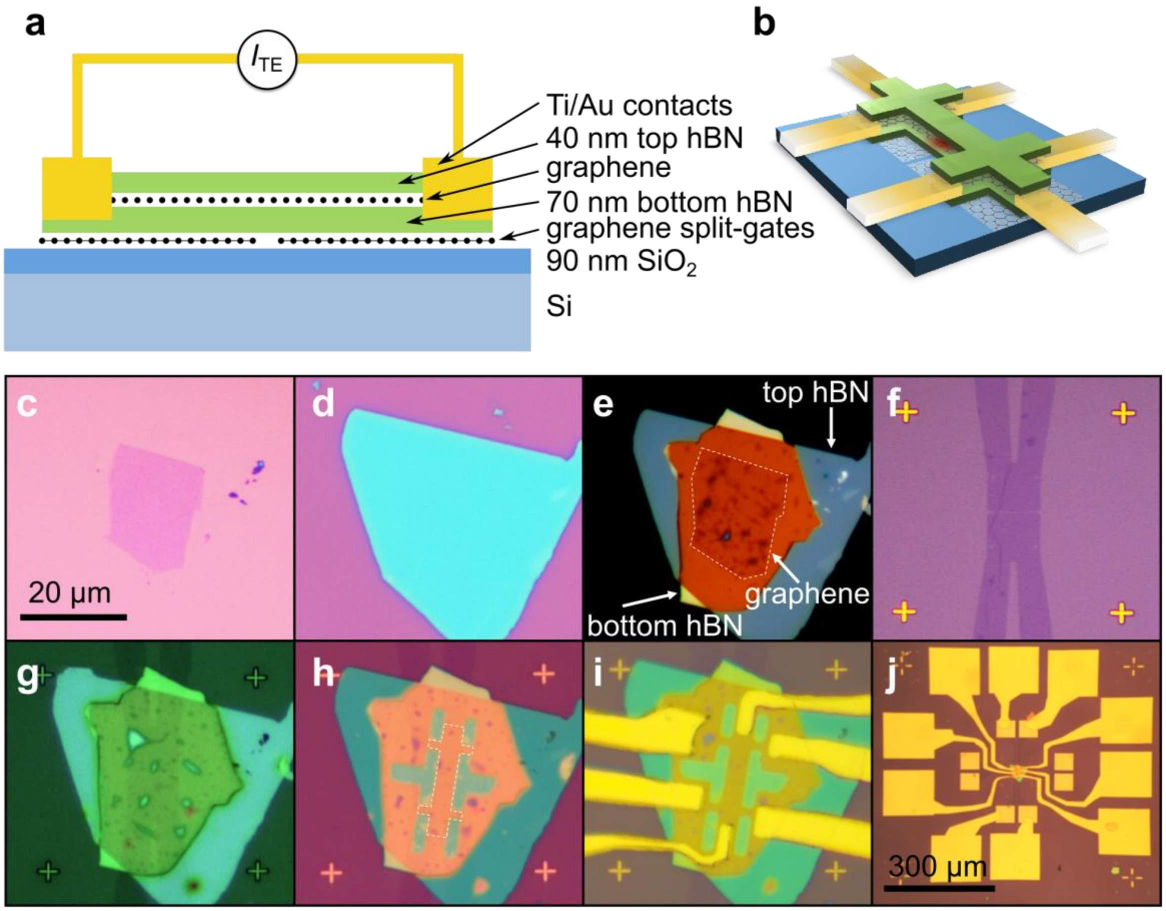

Suppl. Fig. 6: Hall-bar split gate device fabrication. (**a**) Schematic side view showing the hBN-graphene-hBN stack placed on CVD graphene split-gates laying on a Si/SiO_2 substrate. (**b**) 3D schematic of the Hall-bar geometry. (**c-j**) Optical microscope images of the fabrication steps: Exfoliation of graphene (**c**) and hBN (**d**). The stack of hBN-graphene-hBN (**e**) is placed on split-gates (**f**, **g**). We etch parts of the top hBN and graphene layer to create a Hall bar (**h**). Finally, we evaporate Ti/Au contacts (**i**, **j**). Panels (**c**)-(**i**) have the same scale. Figure adapted from Ref. [5], where more fabrication details can be found.

Supplementary Figure 7.

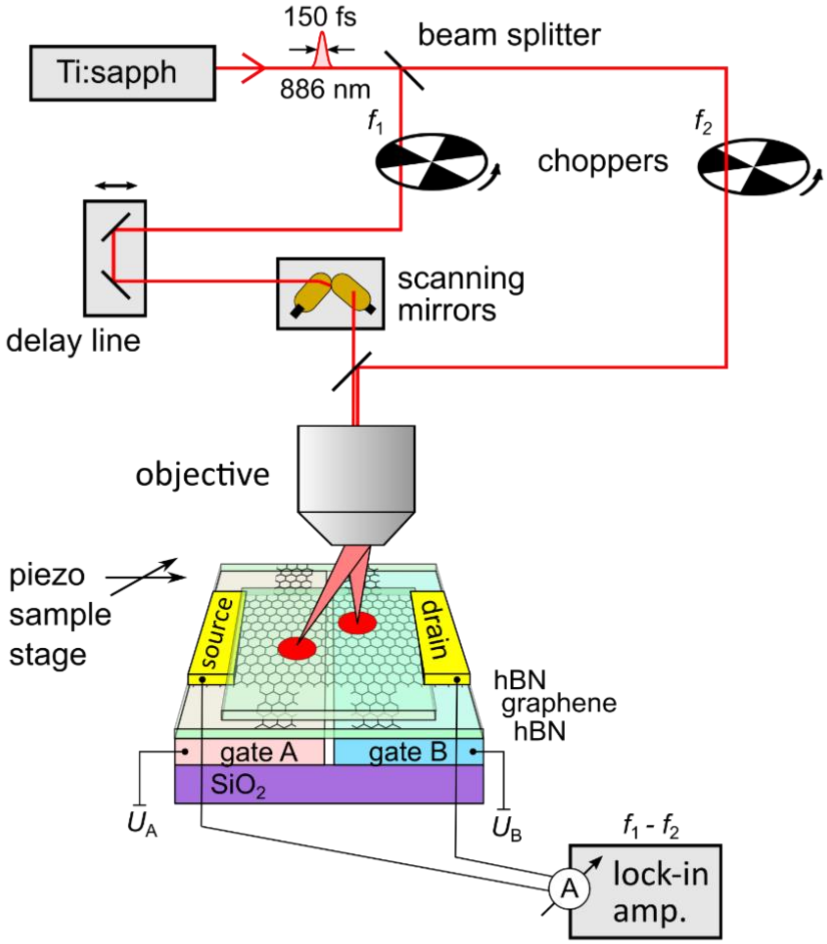

Suppl. Fig. 7: Schematic of experimental setup. The Ti:sapphire laser (*Coherent* Mira 900, λ = 886 nm, 76 MHz rep. rate) is split into two paths with a beam splitter. Each beam is modulated with an optical chopper (*Newport* New Focus 3501 and *Thorlabs* MC2000B). The temporal offset is controlled by a mechanical delay line. The pulses' spatial offset and positioning with respect to the sample is achieved by piezo sample scanning (*Mad City Labs* Nano-LPS100) and galvo mirror scanning (*Thorlabs* GVS012). The beams are combined and focused by a 40x/NA 0.6 objective lens (*Olympus* LUC Plan FLN). The source-drain current is demodulated at 10^6 V/A gain by a lock-in amplifier (*Stanford Research Systems* SR830) at the difference frequency of the individual chopper frequencies to extract the differential thermoelectric current ΔI_{TE}. The prime integer ratio of 7 to 5 avoids interference of harmonics. Additionally, this technique has the advantages of avoiding low frequency noise as well as isolating the signal of interest from the background TE current, which is useful for experimental signal optimization.

Supplementary Figure 8.

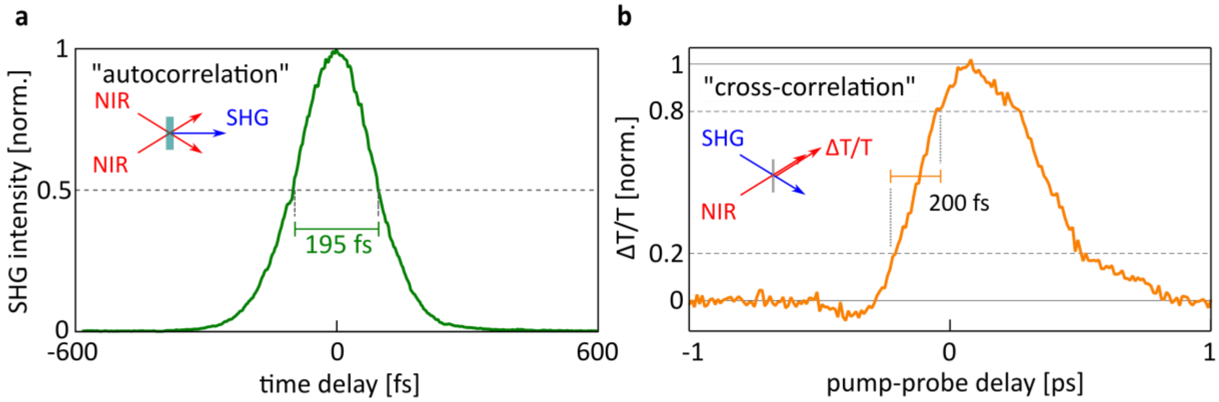

Suppl. Fig. 8: Temporal resolution. (**a**) Autocorrelation of the NIR beam before entering the microscope. (**b**) Transient transmission on CVD graphene, with a frequency doubled pump and NIR probe beam. The 20-80 % rise time of the microscope in the sample plane shows a 200 fs time resolution.

Supplementary Figure 9.

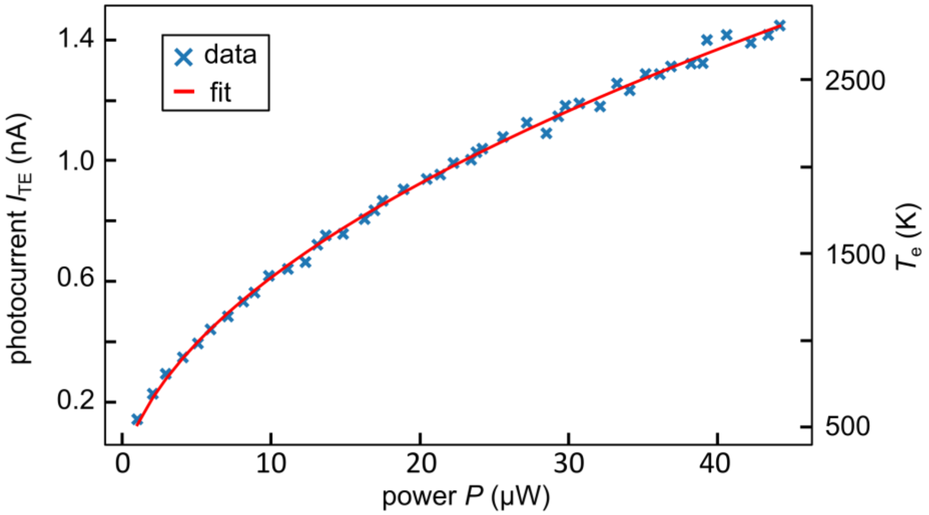

Suppl. Fig. 9: Extraction of electron temperature. The measured thermoelectric current I_{TE} as a function of incident (single) laser power on the junction region. The sub-linear fit to the data is used to estimate the electron temperature (right vertical axis), using $I_{TE}(P) = a\sqrt[2]{T_0^2 + bP} - T_0$, where T_0 = 293 K. The only adjustable parameters are *a* and *b*. The resulting curve (with b = 1.7 $\cdot 10^5$ $K^2/\mu W$) allows us to convert the observed photocurrent into peak electronic temperature T_e (right axis). The same model is used for the spatial distribution of the electron heat in the spatiotemporal simulations (see Eq. S2). Using the electron heat capacity for graphene[6], $\gamma = \frac{2\pi}{3}\frac{k_B^2 E_F}{(\hbar\, v_F)^2}$, with $E_F \approx 0.2$ eV for our experimental conditions, we calculate an experimental pulse energy per unit area and power of $\frac{\Delta Q}{P} = \frac{\gamma b}{2} = 9.8 \cdot 10^{-5}\ \mathrm{J/(m^2 \mu W)}$. We obtain the same value using $\alpha\eta E_{\mathrm{pulse}}/P$, using the beam area, laser repetition rate, heating efficiency η = 80%, and an absorption coefficient α of 0.9%. This absorption is a very reasonable value, taking into account the 285 nm SiO_2 layer and the reflection at the oxide-silicon interface, leading to reduced absorption for an incident wavelength of 886 nm[7].

Supplementary Figure 10.

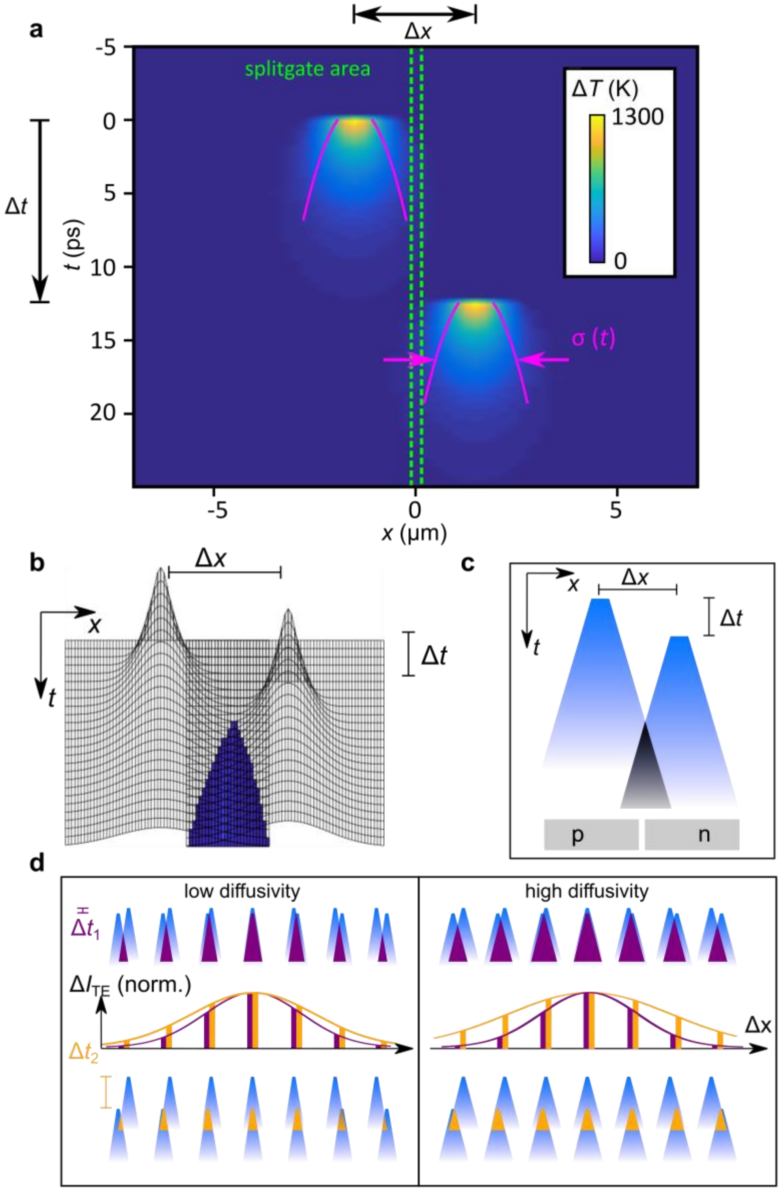

Suppl. Fig. 10: Spatiotemporal heat simulation. (**a**) The total heat profiles are shown as a function of the spatial and temporal lab-coordinates *x* and *t*. Here, a particular set of the variable spatial offset Δ*x* and pump-probe delay Δt is shown. The purple lines show the approximate evolution of σ(*t*) of the two heat pulses. The position of the split-gate (p-n junction) is shown as a green dashed rectangle. (**b, c**) Two representations, highlighting the interaction region due to overlap of the two profiles of heat in space and time, contributing to the differential TE current signal. (**d**) Schematic showing overlapping heat for various spatial offsets Δ*x*, two different pulse delays $\Delta t_1 < \Delta t_2$, for the case of low and high diffusivity, respectively to illustrate the different broadening of the differential TE current signal ΔI_{TE}.

References:

1. Tielrooij, K. J. *et al.* Out-of-plane heat transfer in van der Waals stacks through electron-hyperbolic phonon coupling. *Nat. Nanotechnol.* **13**, 41–46 (2018).
2. Gabor, N. M. *et al.* Hot carrier-assisted intrinsic photoresponse in graphene. *Science* **334**, 648–652 (2011).
3. Hofer, L. R., Dragone, R. V. & MacGregor, A. D. Scale factor correction for Gaussian beam truncation in second moment beam radius measurements. *Opt. Eng.* **56**, 043110 (2017).
4. Woessner, A. *et al.* Electrical detection of hyperbolic phonon-polaritons in heterostructures of graphene and boron nitride. *npj 2D Mater. Appl.* 1–5 (2017).
5. Hesp, N. C. H. Hot-carrier dynamics in hBN-encapsulated graphene. *ICFO - The Institute of Photonic Sciences* (ICFO - The Institute of Photonic Sciences, 2016).
6. Shi, S. F. *et al.* Controlling graphene ultrafast hot carrier response from metal-like to semiconductor-like by electrostatic gating. *Nano Lett.* **14**, 1578–1582 (2014).
7. Tielrooij, K. J. *et al.* Generation of photovoltage in graphene on a femtosecond timescale through efficient carrier heating. *Nat. Nanotechnol.* **10**, 437–443 (2015).
